# The Influence of Auditory Cues on Bodily and Movement Perception

**DOI:** 10.3389/fpsyg.2019.03001

**Published:** 2020-01-17

**Authors:** Tasha R. Stanton, Charles Spence

**Affiliations:** ^1^Pain and Perception Lab, IIMPACT in Health, The University of South Australia, Adelaide, SA, Australia; ^2^Neuroscience Research Australia, Randwick, NSW, Australia; ^3^Crossmodal Research Laboratory, Department of Experimental Psychology, University of Oxford, Oxford, United Kingdom

**Keywords:** auditory, perception, multisensory integration, body perception, movement, emotional valence

## Abstract

The sounds that result from our movement and that mark the outcome of our actions typically convey useful information concerning the state of our body and its movement, as well as providing pertinent information about the stimuli with which we are interacting. Here we review the rapidly growing literature investigating the influence of non-veridical auditory cues (i.e., inaccurate in terms of their context, timing, and/or spectral distribution) on multisensory body and action perception, and on motor behavior. Inaccurate auditory cues provide a unique opportunity to study cross-modal processes: the ability to detect the impact of each sense when they provide a slightly different message is greater. Additionally, given that similar cross-modal processes likely occur regardless of the accuracy or inaccuracy of sensory input, studying incongruent interactions are likely to also help us predict interactions between congruent inputs. The available research convincingly demonstrates that perceptions of the body, of movement, and of surface contact features (e.g., roughness) are influenced by the addition of non-veridical auditory cues. Moreover, auditory cues impact both motor behavior and emotional valence, the latter showing that sounds that are highly incongruent with the performed movement induce feelings of unpleasantness (perhaps associated with lower processing fluency). Such findings are relevant to the design of auditory cues associated with product interaction, and the use of auditory cues in sport performance and therapeutic situations given the impact on motor behavior.

## Introduction

Our perception of our own bodies and our experience of the world around us is fundamentally multisensory in nature (Stein and Meredith, 1993; Driver and Spence, 2000). For example, we see and feel ourselves being gently stroked. Or, in contrast, we experience the jolting sensation of a braking car combined with the sickening sound of tires skidding across the road’s surface. The richness of such multisensory experiences are often taken for granted due to the seamless integration of numerous different sensory inputs. The brain constantly integrates, prioritizes, and filters numerous different sources of incoming sensory information, combining them with the aid of prior knowledge and experience, in order to create a unique perception – namely, a perceptual inference – concerning our body and the environment that surrounds it (Knill and Richards, 1996; Kersten and Yuille, 2003; Ernst and Bülthoff, 2004). This process is dynamic, with perceptual inferences continuously and rapidly being updated in order to allow for adaptive responses to changing bodily properties, or to an environment that is changing (Ernst and Bülthoff, 2004). Moreover, as highlighted in the above examples, the integration of sensory inputs also provides information concerning meaning, influenced by the valence of the stimuli, which then guides appropriate action (primarily conceptualized in terms of approach vs. avoidance). Together, these dynamic adaptations are critical to survival.

Much of the perceptual inference that is relevant to ‘us,’ as individuals – namely, our perception of our own body and our interaction with the environment that surrounds us – involves movement. For example, the movement of a limb, through sensorimotor feedback, helps to shape the mental representations that underlie the perceived length of our limbs (Longo and Haggard, 2010; Proske and Gandevia, 2012). Such knowledge may be crucial in those situations in which our movement might result in harm, such as when reaching to put a log on the burning fire. Furthermore, it is movement that allows us to interact with the environment. In these situations, perceptual inferences from sensory input generated by movement allow us to experience what we are touching, such as the roughness of a surface, as well as to determine its pleasantness or unpleasantness (i.e., its emotional valence) (McGlone et al., 2014). These inferences then inform our consequent motor behavior. For example, how much pressure should we apply in order to touch a rough surface comfortably? What situations should we stay in (because they are pleasant) and which situations should we try to extract ourselves from (because they are unpleasant)? Thus, an individual’s perception and their emotional responses during movement-related activity may well have a number of important implications for a variety of fields. For example, the perception of movement is likely to be of relevance in the treatment of those with movement-related painful conditions, for whom the perception of danger is inappropriately generalized to safe movement situations (Meulders et al., 2015a, b). Our perception of movements and their emotional sequelae may also be critically important for product design such as when the sensory input provided when consumers interact with products is altered to produce a particular desired auditory feedback (Berger et al., 2006; Spence and Zampini, 2006; Wang and Spence, 2019), or for virtual reality (VR) applications where sensory input can be used to heighten the immersive virtual experience (Gallace et al., 2012; Ho et al., 2013).

By now, it is well-established that the integration of visual, tactile, and proprioceptive information plays a key role in updating how we perceive our own body, its movement, and the environment we interact with (Maravita et al., 2003). In contrast, relatively little research has explored the contribution of *auditory cues* to the perceptual inferences that are made during movement-related activity. The last few years have seen a growing interest in audio-motor interactions, particularly in their effect on bodily perception and motor performance. Improved motor performance during development, athletic training, and rehabilitation is underpinned by motor learning. Given that motor learning is based upon motor perception and multisensory representations of action, including audition (Shea et al., 2001), a review of these new studies investigating what might be termed audio-motor interplay is timely and may well have significant ramifications for both training and therapeutic purposes.

Investigation into auditory influences on perception during movement is inherently relevant. After all, almost every bodily movement gives rise to some sort of auditory feedback that provides potentially useful information concerning the movement and providing information about body position (for example, the sound of footsteps during walking). These self-produced sounds are known to be represented in the action-recognition system (Aglioti and Pazzaglia, 2010). For example, neurophysiological evidence in monkeys shows that neurons in the premotor cortex discharge both when a movement is performed as well as when a monkey hears a sound corresponding to that movement being performed (Kohler et al., 2002; Keysers et al., 2003). Similarly, neuroimaging work in humans has revealed that activation within the ventral premotor cortex occurs both during movement and when listening to the sound of that movement (Gazzola et al., 2006). It has been theorized that during movement, an internal representation of the movement is created that allows an individual to determine, using movement-induced sensory feedback, whether the actual movement matches the intended one or not (Wolpert et al., 1995). Transcranial magnetic stimulation (TMS) and functional neuroimaging studies support such an idea, showing that an internal representation of movement exists that is evoked solely by the *sound produced by that movement* (Wilson et al., 2004; Pulvermuller et al., 2006; D’Ausilio et al., 2009). In addition to self-produced sounds, subtle auditory cues that we may be unaware of are often associated with, and can influence, our actions and behaviors (Haley and Fessler, 2005). Together, this supports the potential for profound auditory influences on movement that may stem from varied auditory sources, including those that we may or may not be consciously aware of.

Here, it is also pertinent to consider audio-tactile interactions during self-generated movement; that is, how auditory information generated by tactile contact could impact our perception of our own movement and of the environment that we happen to be interacting with. Such interactions are relevant to consider given physiological, behavioral, and neuroanatomical links between these two senses (von Békésy, 1928, 1957, 1959a,b; Yau et al., 2009; Olson et al., 2012). For example, the receptor organs for both touch and audition depend upon the mechanical displacement of receptors to encode physical stimuli as neural signals. Thus, both auditory and tactile input from self-generated movement provide information about the mechanical energy produced by said movement. Both modalities are also frequency dependent (Yau et al., 2009) which raises the possibility of systematic perceptual interactions, given that the more so-called amodal properties shared by different modalities, the more likely the brain is to attribute them to a common source (Stein and Meredith, 1993). For example, auditory stimuli affect the perception of somatosensory vibrations only when provided at the same or similar frequency (Ro et al., 2009) and this extends to complex, higher-order representations [e.g., tactile sweep direction perception is not influenced by auditory stimuli if provided at a different absolute frequency (Crommett et al., 2019)]. Such findings occur despite temporal frequency matching judgments [same/different] of audiotactile pairs being *least* accurate for small discrepancies between stimuli (Occelli et al., 2009). If two senses detect very highly correlated information (e.g., vision and touch detecting object shape or audition and touch stimulated by the same kind of energy), then stronger coupling priors occur, with the result being increased binding (Parise, 2016; Chen and Spence, 2017). That is, having overlapping or shared mechanical stimulus in the environment may increase integration. Last, neural links between feeling and hearing have been supported by functional neuroimaging that has revealed extensive ipsilateral connections between the primary auditory and somatosensory cortices (Ro et al., 2013). Taken together, current evidence provides compelling support for the existence of crossmodal interactions between sound and touch. In fact, there is evidence to suggest that in some situations, auditory input may be more heavily weighted than tactile input in shaping perception (Huang et al., 2012), although such interactions are likely situation- and task-dependent – for example, see Occelli et al. (2011a) for differences in audiotactile interactions between front and rear space (i.e., surrounding the body).

In addition to self-produced sounds induced by movement or touch, there are also associative pairings between movement and sound that provide information about the action needed, or else performed. For example, the report of a gun to signal the start of a race or the buzz of an alarm clock early in the morning all provide input regarding the action needed. In contrast, the sound of a ball hitting the ground, or of a piano note to a musician, provide relevant feedback concerning the action just performed. These associative pairings can occur even when one does not perform the movement oneself, i.e., merely when observing someone else’s movement (Launay et al., 2016), or with novel auditory cues that are typically unrelated to the movement performed (e.g., a low frequency [low pitch] tone) (McNamara et al., 2008). Indeed, neuroimaging findings have shown that even unrelated auditory cues may become associated with the neural substrates underlying the motor action (i.e., movement that is paired with the auditory cue) (McNamara et al., 2008). Lastly, there are also movement-relevant associations between spatial features of a stimulus, non-naturalistic sounds (e.g., pitch/intensity), and emotional states (Tajadura-Jiménez et al., 2010). For example, higher-pitched sounds are perceived to be positioned higher on a vertical axis than are lower-pitched sounds (Pratt, 1930; Roffler and Butler, 1968). Meanwhile, sounds that ascend in pitch are associated with both elevation (Maeda et al., 2004; Sadaghiani et al., 2009; Deroy et al., 2018) as well as visual expansion (Eitan et al., 2014), while descending-pitch sounds are associated with descent (Maeda et al., 2004; Sadaghiani et al., 2009; Deroy et al., 2018) as well as visually shrinking size (Eitan et al., 2014). Additionally, sounds that rise in pitch are perceived as approaching the body and, when unpleasant, such approaching sounds result in a significantly more intense, negative emotional response than those evoked by sounds that are perceived as receding (i.e., falling pitch) (Tajadura-Jiménez et al., 2010). Similarly, sounds that increase in intensity (dB) are perceived as looming (moving toward the body), are perceived as more unpleasant, and induce increased defensive responses (skin conductance response) than sounds that decrease in intensity (perceived as receding) (Bach et al., 2009). Despite these compelling findings, less is currently known about how such spatially relevant auditory cues influence, or impact, an individual’s movement-related activity, including the perception of one’s own body and its movement. Clearly, a more nuanced understanding of how the physical and emotional perception of movement-related activity is shaped will be critical to guiding a fundamental understanding of perceptual inference and to translate these findings into training and clinical environments.

An individual’s perceptual inferences are dynamically updated on the basis of the available sensory information (Ernst and Bülthoff, 2004). These continual updates provide a unique way in which to evaluate the influence of auditory cues on movement-related activity: namely, by intentionally manipulating incoming auditory input (making it inaccurate in terms of context or timing, for example) in order to determine its influence on perception and behavior. Importantly, action can sharpen the fidelity with which sensory signals (such as audition) are represented (Yon et al., 2018) thus suggesting that online, recursive modulation of audio-motor interactions (and therefore performance) can occur. These bi-directional influences between audition and action support the potential for profound influences on perception. Given the rapidly growing literature on auditory influences on movement and body perception (Tajadura-Jiménez et al., 2015a, 2016, 2017b, 2018; Stanton et al., 2017), a review is clearly warranted. No such published review currently exists in this space as multisensory research has tended to focus on visual influences on body and movement perception, for example, see Moseley (2005); Moseley et al. (2008), and Stanton et al. (2018), and previous reviews of audiotactile interaction are based on passive tactile stimulation – e.g., see Kitagawa and Spence (2006) and Occelli et al. (2011b).

While the same multisensory integration processes may well be expected to operate no matter whether veridical (accurate) or non-veridical (inaccurate) auditory input is provided as part of multisensory experiences, the ability to detect the impact (or relative contribution) of each sense when they provide a slightly different message, so to speak, is greater. For example, considering Bayesian inference (Vilares and Kording, 2011), a noisy sensory input that *challenges* the prior (e.g., a different sound than we would typically expect to hear with movement), would stand a greater chance of shifting the posterior (perception) than if the sound typically paired with movement is provided. Thus, studying the combination of non-veridical inputs may make any perceptual shifts easier to detect. However, perhaps more importantly, the relevance of sensory incongruence (i.e., non-veridical auditory input) is not limited in scope to lab-based experimental manipulation: it also has important implications for the real world. After all, there are numerous situations in which, despite identical multisensory input, incongruent audio-visual impressions can occur. For example, during thunder and lightning, despite synchrony of light and sound being emitted, we typically see lightning before hearing the associated thunder, due to physical differences in the speed of modality transmission through air (light is faster; see Spence and Squire, 2003). There are also biophysical modality differences in the speed of transduction: mechanical transduction at the ear is faster than chemical transduction at the retina. Thus, at an optimal distance (∼10 m) the physical and biophysical differences cancel each other out, and arrival of visual and auditory input at the brain is synchronous. However, many audiovisual events are perceived as synchronous despite not being experienced at the optimal distance (thus are actually temporally incongruent in the brain). Other examples of natural incongruence between the senses include inherent auditory and visual differences in flicker/flutter rate perception (Welch et al., 1986) and in spatial localization (Pick et al., 1969). Finally, discordant afferent inputs are also recalibrated (or suppressed) to confirm a predicted state of the world during self-movement: actions (e.g., pressing a button) and feedback (delayed audio beep) can be perceived to be closer in time (Haggard and Chambon, 2012; Khalighinejad and Haggard, 2016). Such findings suggest that our brain often has to work with multisensory inputs, that are, in some sense at least, incongruent, and yet often it integrates them successfully.

**FIGURE 1:**
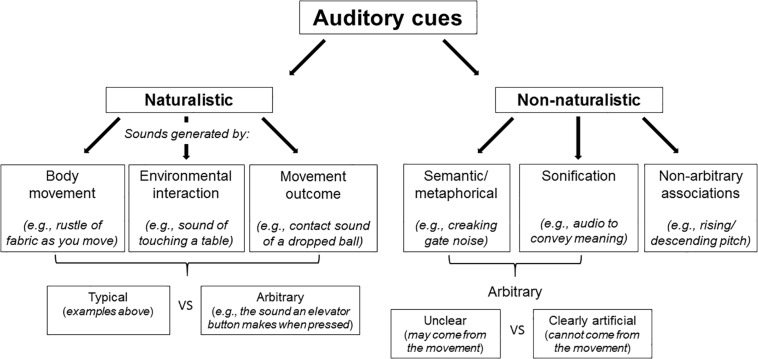
Classification of auditory cue types.

The aim of the present review is therefore to summarize the available evidence concerning the influence of *non-veridical* (i.e., inaccurate) auditory cues on the perception of: (i) the body; (ii) movement; (iii) the environment that is interacted with, as well as considering the effect on emotion (e.g., pleasantness) that such pairings may produce. This review specifically aims to determine whether there are systematic influences on perception that are dependent upon the type of non-veridical auditory input. Auditory cues can be inaccurate in numerous ways. For example, cues can be too loud, too quiet, they may come from the wrong direction, be delayed, or perhaps distorted in some way. Finally, this review will also consider the influence of auditory cues on movement itself. These findings will be discussed based on the context of the sound (see Figure 1) – that is, whether or not the auditory cues are naturalistic (i.e., relevant to the body and to movement, or its outcome) or non-naturalistic/artificial (e.g., sounds with semantic associations with movement, or its outcome, or else auditory cues that are unrelated) (Walker-Andrews, 1994). Naturalistic cues can either be arbitrary (e.g., the sound that occurs when you press a button – differs based on what button you press) or typical, such as the sound of a ball hitting the ground after dropping it (Walker-Andrews, 1994). Non-naturalistic cues can be non-arbitrary, such as a rising pitch associated with movement of an object toward you, or arbitrary, the latter of which may also be clearly artificial (e.g., pairing of a sound with movement that realistically cannot come from that movement) or unclear (e.g., the sound may well come from that movement). This review also includes discussion of those studies that use sonification, referring to the use of altered auditory input, i.e., non-speech audio, to convey information. Given the large field of sonification research (for example, see Bevilacqua et al., 2016; Schaffert et al., 2019), this review focuses on sonification that is temporally or contextually discordant with movement, that is, when it is intentionally unmatched to the movement performed.

## Influence of Auditory Cues on Perception of the Body

Auditory cues provide important information concerning our body. Such cues can include the sound that results from movement of the body itself or that result from our interaction with the environment, which, in turn, allows us to make perceptual inferences about the state of our own body. Auditory cues can also provide useful information about our bodily properties via feedback corresponding to the effects or outcomes of our action – particularly, when objects are involved. The next section discusses the evidence that has been published to date concerning the influence of such auditory cues on body perception (see Table 1 for summary of findings).

**TABLE 1:** Summary of the effects of non-veridical auditory cues on body perception.

	**Auditory Dimension**	**Perceptual Dimension**	**Findings**	**Studies**
**Auditory cue type: Naturalistic (typical)**	**Increasing intensity (dB), of:**			
	• Hand rubbing auditory feedback	Skin perception (Parchment skin illusion)	Skin feels rougher and dryer	Jousmäki and Hari, 1998
	**Amplifying high frequency components, of:**			
	• Hand rubbing auditory feedback	Skin perception (Parchment skin illusion)	Skin feels rougher and dryer	Jousmäki and Hari, 1998; Schiller, 1932; Guest et al., 2002
	• Walking auditory feedback	Body weight	Body feels lighter (↓ perceived weight)	Tajadura-Jiménez et al., 2015a
	**Audio incongruence (vs. actual movement/touch), of:**			
	• Hand rubbing auditory feedback	Skin perception (Parchment skin illusion)	No alteration in skin perception when sound not matched to movement	Guest et al., 2002
	• Auditory feedback of a dropped ball (takes more time or less time to hit the ground than it should)	Body height	↑ Height with audio delay of a dropped ball (takes longer for ball to hit the ground than it should) No change in height with audio advance (takes less time for ball to hit the ground than it should)	Tajadura-Jiménez et al., 2018
	• Sound of paintbrush strokes	Hand ownership (rubber hand illusion)	↓ brush strokes sounds are not paired with tactile paintbrush strokes. ↑ when auditory and tactile are paired.	Radziun and Ehrsson, 2018
	• Sound of finger tapping	Hand ownership (rubber hand illusions)	↓ownership when sound not paired with participant’s passive touch of rubber hand (+ researcher touch of real hand) ↑ownership when paired	Radziun and Ehrsson, 2018
	**Spatial incongruence (vs. actual location), of:**			
	• Sound of finger tapping on table	Arm length (via tactile distance estimation task)	↑ perceived arm length with manipulation of spatial distance of sound (2x the distance of actual sound origin) No effect of 4x the distance of actual sound origin	Tajadura-Jiménez et al., 2012 Replication: Tajadura-Jiménez et al., 2015b
**Auditory cue type: Non-naturalistic**	**Sounds with semantic meaning**			
	• Creaky door sound • Gentle whoosh sound	Back stiffness (via surrogate of force perception)	↑ by “creaky door” sound ↓ by gentle “whoosh” sound	Stanton et al., 2018
	• Sound of marble being hit with a hammer	Material properties of the hand (Marble hand illusion)	Sound of marble hit with hammer paired with hammer tapping the skin: ↑ feelings of hand stiffness & heaviness; felt less sensitive and less natural when sound paired with a hammer tapping the skin (vs. audio delay)	Senna et al., 2014
	**Non-arbitrary associations**			
	• Rising pitch (=longer)	Finger length	↑ length of finger when self-pull on one finger paired with a rising pitch sound	Tajadura-Jiménez et al., 2017b
	**Audio incongruence (vs. actual movement/touch), of:**			
	• Sound of a metronome	Space ownership (invisible hand illusion)	↓ proprioceptive drift when metronome not timed with stroking the real hand and stroking an empty area of space ↑ proprioceptive drift when audio-tactile paired	Darnai et al., 2017
	• Virtual xylophone	Hand ownership (rubber hand illusions)	↓ embodiment ratings when musical output not paired with visuotactile cues ↑ embodiment ratings when musical output paired with visuotactile cues	Choi et al., 2016

Providing naturalistic, but non-veridical, body- and movement-relevant auditory cues alters people’s perception of the material properties of their own body. Specifically, previous work has evaluated the effect of altering auditory feedback while people rub their hands together (Jousmäki and Hari, 1998; Guest et al., 2002). Originally explored by Schiller (1932), contemporary research reveals that increasing the average intensity (Jousmäki and Hari, 1998) or amplifying just the high–frequency components (Jousmäki and Hari, 1998; Guest et al., 2002) of the sounds emitted during hand rubbing modified the perception of the qualities of an individual’s skin: the palmer skin surface feels rougher and drier (hence the name, ‘the parchment skin illusion’) than when hand rubbing sounds were not intensified/amplified. Critically, delaying auditory feedback of the hand rubbing was shown to reduce the magnitude of this crossmodal illusion (Jousmäki and Hari, 1998; Guest et al., 2002), thus suggesting that the temporal pairing and synchronization of that sound with movement is key to evoking the illusory rougher/drier sensation. Such findings support the presence of a crossmodal modulation of the incoming sensory input, given that temporal coincidence of multisensory input is a key feature of crossmodal binding/multisensory integration (Wallace et al., 1996).

**FIGURE 2:**
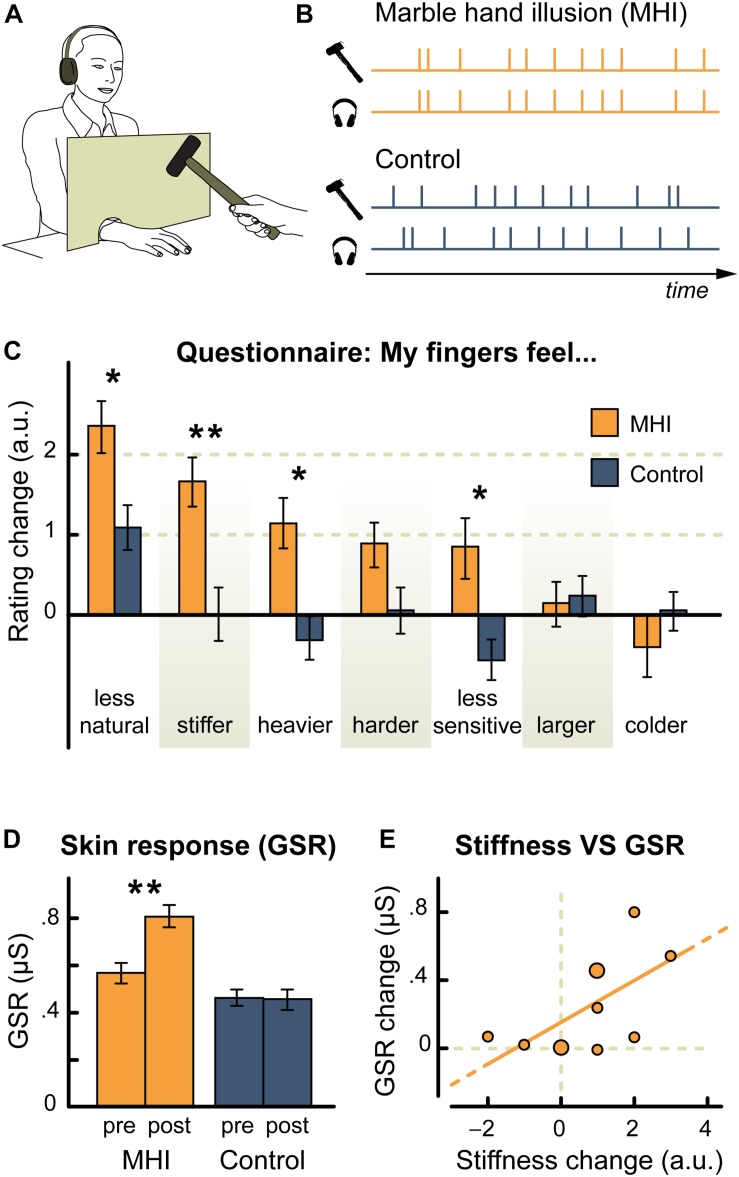
Experimental set-up and results from Senna et al.’s (2014) marble hand illusion Experiment 1. **(A)** Experimental set-up; **(B)** Experimental conditions of temporally synchronous sound and skin tap (marble hand illusion) or asynchronous sound and touch (control); **(C)** Results for perceived finger properties assessed via questionnaire (Mean change [post- minus pre-testing] ± standard error of the mean); ^∗^*p* < 0.05; ^∗∗^*p* < 0.01; **(D)** Results for arousal to a threatening stimuli as measured using galvanic skin response (GSR), with findings showing an increase in arousal for the marble hand illusion condition but not the control condition (mean and standard error of the mean shown); **(E)** Relationship between perceived hand stiffness and mean arousal (GSR) for the marble hand illusion condition. A positive significant correlation (Pearson’s *r* = 0.6, *p* = 0.02) was found between changes in perceived hand stiffness and changes in arousal (larger dots represent two points falling in close proximity). [Reproduction of Figure 1 of Senna et al. (2014). Reproduced via the Creative Commons Attribution (CC BY) License]. ^∗^Color not needed for publication.

Similarly, pairing non-veridical auditory cues with mechanical pressure/touch applied to the body also results in the updating of perceived material properties of the body. First, pairing non-naturalistic auditory cues that have semantic associations with stiffness (think here only of the sound of a creaky door vs. the sound of a gentle ‘whoosh’) to pressure applied to the back, modulates the perception of back stiffness (as measured using force magnitude assessment). This modulation is dependent upon the nature of the sound presented (Stanton et al., 2017). A ‘creaky’ door sound was found to increase perceived back stiffness, while a gentle whooshing sound decreased perceived stiffness as did repeated application of a creaky sound that reduced in volume over time (Stanton et al., 2017). A second study evaluated the effect of temporally pairing gentle contact on the hand (using a small hammer) with the sound of a hammer (Senna et al., 2014). The auditory cues progressively changed from an accurate sound of a hammer hitting the skin to an inaccurate sound of hitting a piece of marble instead (see Figure 2) (Senna et al., 2014). Over time, this temporally synchronous pairing resulted in an increased perception of hand stiffness, with participants also reporting that their hand felt heavier, less sensitive, and somehow ‘unnatural’ as compared to an asynchronous control condition (Senna et al., 2014). Intriguingly, participants also displayed an enhanced galvanic skin response (GSR) to threatening stimuli (Senna et al., 2014). Taken together, such findings suggest that auditory cues are continually integrated in order to update the perception of the body’s material properties. Furthermore, these findings also suggest that body perception can be altered, even if the auditory cues are not body-related but rather have some kind of semantic associations with known material properties, given that the auditory cues are temporally and/or spatially paired with either sensory input or motor output.

**FIGURE 3:**
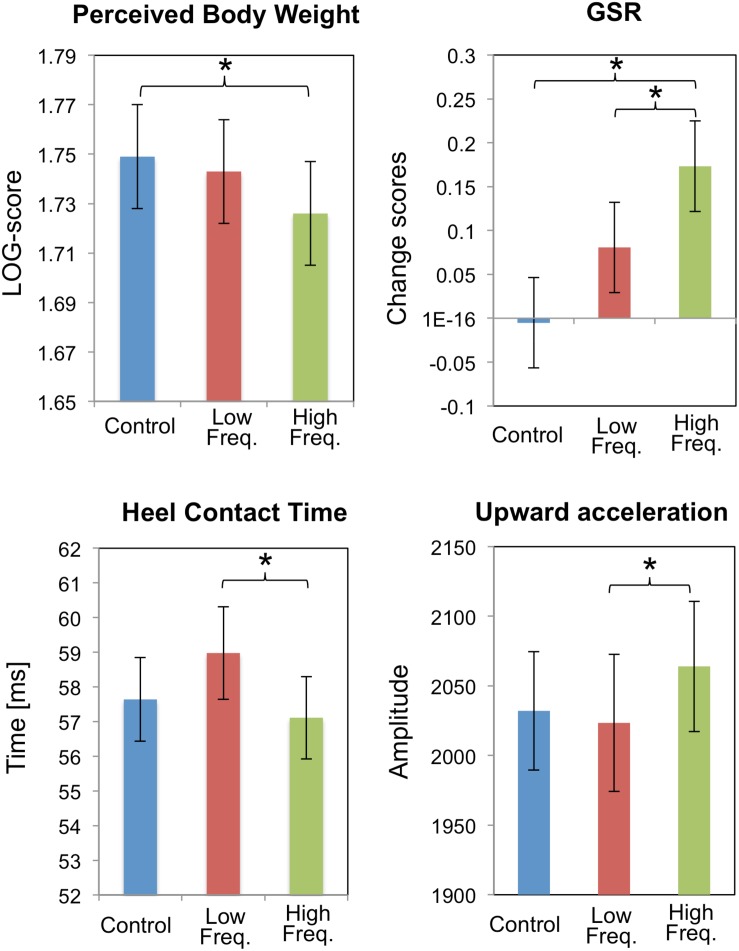
Results from Tajadura-Jiménez et al.’s (2015a) experiment highlighting the consequences of frequency manipulation (normal, low, high) of the sound of footsteps while walking. They explore the effects of this manipulation on perceived body weight, galvanic skin response (GSR), heel contact time (milliseconds) during walking, and acceleration of the foot’s upward movement during walking. All values are mean ± standard error. ^∗^Denotes a significant difference between means. [From Figure 4 of Tajadura-Jiménez et al. (2015a). Reproduced with permission of the Association for Computing Machinery in the format Republish in a journal/magazine via Copyright Clearance Center].

Auditory cues impact not only the perceived material properties of the body, but also the perception of the size of the body itself. For example, when the frequency of the sounds of self-produced footstep (i.e., naturalistic) were altered, people’s perception of their own weight changed (Tajadura-Jiménez et al., 2015a). Specifically, shifting footstep sounds to higher frequencies caused participants to perceive their body as being lighter (vs. no sound and low frequency footstep sounds), and such perception was accompanied by increased emotional arousal (GSR) – see Figure 3. Furthermore, such an impact of auditory cues on perceived body size was found to extend to a situation in which unnatural auditory input was paired with self-induced force on the body (Tajadura-Jiménez et al., 2017b). In the latter study, participants used their left hand to pull on the tip of their right index finger. When a rising pitch sound was paired with the pulling of the finger, the participants both felt (self-report) and estimated their finger to be longer than when the pull was paired with a descending pitch or a tone having a constant pitch instead. The authors refer to this as the “auditory Pinocchio effect” (Tajadura-Jiménez et al., 2017b). The perception of finger elongation reported in this study (following the pairing of tactile input with a rising pitch sound) was independent of the orientation of the hand (and of the direction of the pull based on hand position) (Tajadura-Jiménez et al., 2017b). Such findings therefore provide empirical evidence that sounds do not necessarily need to be ecologically or contextually relevant, rather, unnatural sounds that have meaning can induce crossmodal effects that may result in changes to body perception.

Auditory cues providing information about the results of an action – movement-relevant information, in other words – can modulate people’s perception of their own body. According to the results of a recent study, naturalistic sounds that provide information about moving objects alter height perception (Tajadura-Jiménez et al., 2018). The participants in the latter study dropped a ball from shoulder height, with auditory and tactile stimuli that provided information to the participant about when the ball hit the ground. These audio and tactile cues were then altered to manipulate the perceived time it took for the ball to hit the ground. Artificially increasing the latter resulted in a perception of increased body height (i.e., an elongation of the body). Given that the brain predicts the consequences of action (see (Angelaki et al., 2004), for evidence that representations are held of internal models of physical laws of motion), we expect the ball to hit the ground after a certain delay based on how far it is held off the ground. Thus, when the auditory feedback doesn’t match the prediction, it was proposed that the mental representation of body height is altered to make sense of the feedback (i.e., my body must be taller than I thought) (Tajadura-Jiménez et al., 2018). Alternatively, such auditory cues may also feasibly update the spatial position of the body in relation to the environment (e.g., I must be standing on an uneven surface, such as near the bottom of stairs, so the ball has farther to fall). The fact that auditory influences on body spatial position were not assessed in this study means that its potential contribution to perceptual changes cannot be ruled out.

It is unknown whether such proposed alterations in perceived body morphology are perceptual in nature, i.e., result from auditory cue-induced updates to the mental representation of the body, or whether instead they are more decisional in nature, whereby cognitive processes are engaged to over-ride an existing mental representation. Interestingly, perceptual shifts in body height only occurred when the simulated ball drop height was increased but not when ball drop height was decreased (i.e., half height simulated condition) (Tajadura-Jiménez et al., 2018). Such findings suggest that there are limitations to the ability of auditory cues to change the representation and perception of the body. While speculative, it is possible that prior experience influences the unidirectional changes in perceived body elongation (i.e., height) documented here: nearly everyone has experienced being taller through the use of a heeled shoe. Perhaps such ‘taller’ body representations are more easily accessible or result in increased malleability of perceived body height change in that direction. An enhanced ability to *increase* perceived body size (i.e., trunk or limb elongation) might also be predicted based on tool use, whereby external objects are integrated into one’s body schema (Holmes and Spence, 2006; Martel et al., 2016), thus increasing effective limb length. Clearly further work is needed to investigate whether auditory-induced alterations to perceived body size are unidirectional. Given that auditory input has high levels of spatial acuity, future work could evaluate experimental object-drop height conditions at much smaller non-veridical increments (e.g., 90% of normal height, 80% of normal height, etc…). There are surely limits to which a body can feel shorter and experimental conditions using auditory cues consistent with 50% of normal height may potentially be breaching this limit.

Such effects of action on body perception are supported by work showing that naturalistic sounds produced by tapping on a surface with one’s hand, when spatially manipulated to sound as though they were occurring from double or quadruple the distance of the origin of the actual sound alter perceived limb size (as assessed using a tactile distance estimation task) (Tajadura-Jiménez et al., 2012). Exposure to the tapping sounds in the double auditory condition resulted in an increase in the perceived tactile distance on the test arm (vs. veridical condition), congruent with a perceived elongation of the test arm (Tajadura-Jiménez et al., 2012). No changes occurred with an accurate or quadruple condition. Again, the finding that the quadruple condition had no effect on tactile distance perception supports the view that the extent to which body size perception can be altered by auditory cues may have inherent limits. Changes in perceived tactile distance due to spatial manipulation of tapping sounds (suggestive of an elongated arm) were replicated in work that showed that both agency (feeling that the sounds are coming from your tapping) and kinesthetic cues (the actual position of your arm) are important to the effect (Tajadura-Jiménez et al., 2015b).

Finally, recent work has shown that naturalistic auditory cues can influence perceived body ownership. Tested via embodiment illusions such as the rubber hand illusion (RHI), it has been shown that the feeling of ownership over a rubber hand can be strengthened or diminished, respectively, depending upon whether auditory cues (the sounds of brushstrokes) are veridical (matched) or non-veridical (unmatched) with the tactile input. During the RHI, a rubber hand is stroked at the same time and in the same location relative to the body surface (synchronous stroking) as an individual’s real, hidden hand (Botvinick and Cohen, 1998). Such synchronous stroking creates the sense that the rubber hand is one’s own and shifts perception of hand location toward the location of the rubber hand, the latter termed proprioceptive drift (Botvinick and Cohen, 1998). If the sounds of brushstrokes are synchronized with the touch of the rubber and real hand (by paintbrushes), the illusion is strengthened (embodiment ratings and proprioceptive drift) compared with a no-sound condition (synchronous touch, but no auditory cue) (Radziun and Ehrsson, 2018). By contrast, if the auditory input is non-veridical (i.e., not paired with the touch of the paintbrush), the strength of the illusion is weakened relative to the synchronous audiotactile condition and does not differ from an asynchronous visuotactile control condition (no auditory cue) (Radziun and Ehrsson, 2018).

Similar effects of auditory cues on body ownership have also been seen in a motor version of the RHI: with vision occluded, the researcher passively moved the participant’s hand to touch the 2nd knuckle on the rubber hand, while touching the knuckle of their real hand either synchronously or asynchronously (Radziun and Ehrsson, 2018). Auditory cues consisting of the sound of finger taps enhanced the strength of the illusion (leading to increased proprioceptive drift) when applied synchronously, but when auditory cues provided non-veridical (i.e., asynchronous) information, the effect was diminished (Radziun and Ehrsson, 2018). Largely consistent effects on body ownership were seen for non-naturalistic auditory cues. When auditory input (the sound of a metronome) was paired with an invisible hand illusion (stroking the real hand and stroking an empty area of space such that the space is ‘embodied), the effect of the illusion was stronger (in terms of the magnitude of the proprioceptive drift observed) than when no auditory cues were present (Darnai et al., 2017). Additionally, in a motor version of the RHI, whereby VR was used to create a virtual xylophone that provided synchronous visual, tactile, and auditory (musical) input – auditory cues were found to enhance the ratings of embodiment given during this virtual hand illusion (Choi et al., 2016). While these latter two studies (Choi et al., 2016; Darnai et al., 2017) use only veridical auditory cues, their findings provide further support for the effect of sound on body ownership.

### Interim Summary

Taken together, the evidence that has been published over the last decade or so clearly highlights the crossmodal influence that auditory cues have on the perception of the body. Research in which inaccurate sounds have been synchronized with movement and with movement-related outcomes demonstrates that auditory cues can induce profound changes in body perception. Such auditory findings are largely consistent with visual modulations of body perception. Inaccurate visual (or visuotactile) cues of body size/shape rapidly update the perceived size of the body (Gilpin et al., 2015), visual illusions of tool use (achieved using a mirror box set-up) modulate tactile perception on that body part (Miller et al., 2017), and congruent visual and tactile input can induce ownership of a rubber hand (Botvinick and Cohen, 1998) and loss of one’s own hand (via the Disappearing hand illusion) (Newport and Gilpin, 2011). Together, these findings suggest that distortions to the perception of the body can be achieved via numerous and varied sensory sources, including auditory input. Of interest, none of the studies purposefully evaluated the duration of auditory-induced body perceptual alterations, although past research on the theme of visuotactile body illusions supports the temporary nature of these modifications (Lane et al., 2017). Last, it is also relevant to consider that movement-relevant body perception and auditory cues have bi-directional influences: whole body rotation (vestibular input of body rotation) influences auditory percepts, namely sound lateralization, in the direction of rotation (Lewald and Karnath, 2001).

The fact that perceptual changes were induced via the spatial and/or temporal pairing of auditory cues with sensorimotor input suggests that mechanisms of multisensory integration (Stein and Meredith, 1993; Ernst and Bülthoff, 2004; Spence, 2011) may underlie such perceptual effects. Indeed, the maximum likelihood estimation (MLE) approach to cue integration (Ernst and Banks, 2002) would then also suggest that the reliability of the sensory cues (e.g., sound vs. touch) will determine how heavily it is weighted by the brain and thus determine its ability to alter the overall multisensory percept. Exploring those circumstances in which auditory cues are highly reliable, such as when providing information about the force of a tennis ball coming straight at you (Mead and Drowatzky, 1997) for example, would allow one to test the hypothesis that MLE can be used to model body- and movement-relevant perceptual inference. Similarly, causal inference in multisensory perception via Bayesian modeling provides relevant information for perceptual changes based on the combination of prior knowledge/experience with noisy incoming sensory input (Vilares and Kording, 2011). Indeed, priors are independent of current sensory feedback (Beierholm et al., 2009), thus suggesting that unique perceptual shifts can occur based on past experience or knowledge. Such models would predict an effect of tennis expertise in the ability for auditory cues to influence behavior or judgments about movement outcome. While recent work clearly highlights that expert tennis players’ judgments of tennis ball trajectory are influenced by the sound heard (Canal-Bruland et al., 2018), no work has directly evaluated the effect of tennis player expertise (inexperienced vs. expert) when non-veridical auditory input is provided. Past work has focused on the effect of tennis expertise when either accurate or no auditory input is provided (Mead and Drowatzky, 1997).

## Influence of Auditory Cues on the Perception of Movement

Auditory cues paired with movement, when non-veridical, may impact our own perception of the movement that we have just completed. For example, we might perceive that we have not reached as far if we hear a sound that is closer to our body than would have been generated by our actual reach. Despite this possibility, to date, few studies have attempted to evaluate the effect of auditory input of an individual’s perception of their *own movement.* See Table 2 for a summary of findings.

**TABLE 2:** Summary of the effects of non-veridical auditory cues on perceived movement.

	**Auditory Dimension**	**Perceptual Dimension**	**Findings**	**Studies**
**Auditory cue type: Naturalistic (typical)**	**Increasing intensity (dB), of:**			
	• Sound of finger tapping on the table (compared virtual reality vs. real surface)	Perceived ability to tap Perceived strength Perceived force	↓ perceived ability to tap when a quiet tapping sound was paired with tapping movement on real surface (vs. medium/loud) ↑perceived ability to tap, perceived strength, and perceived force for real vs. virtual (medium sound)	Furfaro et al., 2015
	**Amplifying high frequency components, of:**			
	• Walking auditory feedback	Speed of walking	Speed of walking feels quicker	Tajadura-Jiménez et al., 2015a
	**Audio incongruence (vs. actual movement/touch), of:**			
	• Walking auditory feedback	Movement initiation	↓ sense that they had initiated the movement when sound not matched to movement	Menzer et al., 2010
**Auditory cue type: Non-naturalistic**	**Semantic vs. Arbitrary sound:**			
	• Virtual drilling via haptic device, adding white noise or classical music (vs. naturalistic drilling sound)	Perceived drilling depth	No influence of type of sound on perceived drilling depth	Melaisi et al., 2018
	**Audio incongruence (vs. actual movement/touch), of:**			
	• Bespoke melody consisting of high and low tones	Perceived exertion	↑ perceived exertion when music was not matched to movement (passive listening) compared with condition of musical agency (music matched to movement)	Fritz et al., 2013

Two studies investigated the influence of auditory feedback while walking. The first found that when the sound of footsteps (i.e., naturalistic) was altered in frequency, with a high frequency sound inducing a feeling of lightness, participants perceived that they had walked more quickly than during low frequency alteration of footsteps or during a control condition where natural footstep sounds were provided (equally amplified across all frequency bands; see Figure 3) (Tajadura-Jiménez et al., 2015a). The second study demonstrated that when the sounds of footsteps were temporally delayed while walking, participants had a reduced sense that they had initiated the movement (Menzer et al., 2010), which is largely consistent with prior findings from various visuotactile (Botvinick and Cohen, 1998) and visuomotor (Drummer et al., 2009) ownership illusions.

The effect of auditory cues on movement perception when performing a hand movement has also been investigated during real or virtual interaction. For example, when the sound of tapping a real or virtual surface was altered (quiet, medium, or loud tapping sounds), participants perceived that they were less able to tap when the quiet sound was paired with their tapping of the real surface (vs. medium loudness sound) (Furfaro et al., 2015). Tapping on the real surface resulted in perceptions of greater strength, a self-reported greater ability to complete the tapping task, and participants perceived that they applied more force when tapping on a real surface vs. a virtual surface for the medium sound (Furfaro et al., 2015). For most sound conditions, the participants perceived that they were better able to tap (i.e., complete the task) when tapping on a real rather than a virtual surface (Furfaro et al., 2015). Such differences in induced perceptions of movement between the real and virtual surfaces may reflect the congruence of auditory, tactile, and proprioceptive information in the real surface condition vs. relative incongruence in the virtual surface condition (i.e., no tactile input paired with auditory cues). Similarly, it was found that when performing virtual drilling (holding a haptic device), there was no effect of naturalistic, contextually relevant sound (drilling sound) or non-naturalistic sound (white noise or classical music) on the perception of drilling depth (Melaisi et al., 2018).

Lastly, the effect of sound on perceived exertion during exercise has also been extensively explored, but less so when auditory cues are purposefully inaccurate. It has, for instance, been reported that non-naturalistic auditory feedback paired with movement can alter people’s perception of exercise (Fritz et al., 2013). Specifically, musical feedback was created whereby movement of three fitness machines was transmitted to music composition software, to create a unique musical dimension (including low and high frequency sounds to compose a simple melody). This process was interactive such that small movements of each machine resulted in a noticeable musical effect for the participant. When the auditory musical cues were paired with a participant’s movement during fitness training (i.e., manipulating musical agency), perceived exertion was reduced compared with a condition without musical agency (passive listening – no musical agency).

### Interim Summary

To date, there is limited evidence for auditory influences on the perception of movement. What evidence there is suggests that there may be important differences in the effect of auditory cues on movement perception between real and virtual environments. Previous studies support the presence of perceptual differences contingent on the environment: for example, perception of distance differs between real and virtual environments (Napieralski et al., 2011). Such perceptual differences seen in VR may well extend to movement. In the case of VR, altered sensory input (e.g., visual and/or somatosensory) is often present and may uniquely influence perception of movement, that is, in addition to any perceptual changes induced by the inclusion of auditory cues. For example, when comprehending speech in VR, providing incongruent visual input of lips moving (perception of another’s movement) results in impaired comprehension of the auditory speech (Gonzalez-Franco et al., 2017). However, for the exact reason of sensory ambiguity, auditory cues may play an important role in VR. Numerous studies have shown the potential for auditory re-calibration and/or influence on movement and environmental perception with VR. For example, adding auditory cues to VR can improve source localization via cross-modal plasticity, and thus heighten the sense of presence within VR, while avoiding the need for complex individualized calculations (to enable accurate auditory source localization) (Berger et al., 2018). Additionally, recent work has explored the role of echolocation in VR (via self-produced auditory ‘clicks’) to assist with spatial localization, maze completion times, and environment exploration (Andreasen et al., 2018, 2019). Intriguingly, navigating a VR environment ‘like a bat’ allowed some participants to create cognitive spatial maps based on echolocation, with concurrent improvement in performance (Andreasen et al., 2019). Thus non-typical auditory cues may be able to update self-generated movement in VR, although high training levels appear necessary when the information conveyed by auditory input is non-traditional.

The majority of the studies have evaluated short-term effects of auditory influences on movement perception. It would be interesting for both athletic and therapeutic purposes to know how long these effects on movement perception last. Do perceptions of movement (e.g., walking or running speed) merely revert to baseline levels once the modified auditory input is removed? Or do auditory effects result in stable re-calibration of perception, continuing despite the removal of the modified auditory input? If not, it is relevant to consider whether re-calibration could be sustained via processes of mental imagery (visualizing the movement *with* the sound) given the established link between motor imagery, motor representations, and skilled performance (Kim et al., 2017). Additionally, if auditory perceptual re-calibration were to be long-lasting, it would be of interest to understand what is required to ‘normalize’ movement perception. Is additional auditory input contrary to the original auditory cue needed? Or, perhaps normalization of movement perception could also occur through stimulation in another sensory source such as vision. Finally, given that sensory precision changes over the lifespan, particularly in auditory sensitivity (Liu and Yan, 2007), it would also be interesting to explore whether the auditory influences on movement perception differ as a function of age. There is evidence of age-related changes in multisensory integration for audiovisual interactions (Laurienti et al., 2006; DeLoss et al., 2013); whether such changes extend to audiomotor interactions is less clear.

## Influence of Auditory Cues on Movement-Related Touch

Our interactions with objects and with the environment around us are determined by the sensory feedback resulting from the interaction. As such, auditory input can provide key information about the material properties of the surfaces that we interact with. This section will explore whether auditory cues can alter the perceptual inferences following tactile contact via self-generated movement. See Table 3 for a summary of findings for perception of surface texture and Table 4 for perception of surface contact properties.

**TABLE 3:** Summary of the effects of non-veridical auditory cues on movement-related touch: surface texture perception.

	**Auditory Dimension**	**Perceptual Dimension**	**Findings**	**Studies**
**Auditory cue type: Naturalistic (typical)**	**Amplifying high frequency components, of:**			
	• Sound of abrasive surface being touched (by finger)	Perceived surface roughness	Surface feels rougher (vs. veridical sound)	Guest et al., 2002
	**Attenuating high frequency components, of:**			
	• Sound of abrasive surface being touched (by finger)	Perceived surface roughness	Surface feels smoother (vs. veridical sound)	Guest et al., 2002
	**Attenuating low and middle frequency, of:**			
	• Sound of abrasive surface being touched (by finger)	Perceived surface roughness	No effect on perceived roughness (vs. veridical sound) Both sound conditions altered perception of roughness (low particle size felt rougher; high particle size felt smoother) vs. no sound control	Suzuki et al., 2006
	**Addition of auditory cues to:**			
	• Touch of haptic surface (various virtual surface varying in roughness created via Geomagic Touch device; used sound of a fingertip rubbing against sandpaper and sound of a fingertip rubbing against a sheet of copy paper)	Perceived surface roughness	↓ perceived roughness of all surfaces when touch combined with the ‘sandpaper’ sound (vs. ‘copy paper’ sound and no sound condition) ↓ perceived roughness during audio-tactile incongruence, e.g., a very smooth tactile surface when paired ‘copy paper’ sound (vs. no sound)	Etzi et al., 2018
	• Touch of a haptic device (texture display mouse ‘KAT’; used sound of rubbing a piece of sandpaper)	Perceived surface roughness Perceived surface ruggedness Perceived surface denseness Perceived surface ‘prickliness’	↑ perceived roughness when amplifying 30–600 Hz during virtual touch ↑ perceived ruggedness when amplifying 50–300 Hz during virtual touch ↑ perceived denseness when attenuating frequency (all levels), ↓ perceived denseness when amplifying frequency during virtual touch ↑ perceived prickliness with amplification of frequency levels below 100 Hz	Kim et al., 2007
**Auditory cue type: Non-naturalistic**	**Arbitrary sounds – changing loudness (via amplifying/attenuating), of:**			
	• White noise (four levels of loudness in random order) during touch of abrasive paper of different particle size and length	Perceived tactile roughness Perceived length	Altered perception of roughness (low particle size felt rougher; high particle size felt smoother) No effect on perceived paper length No effect of pure tones (1000 Hz) on either outcome	Suzuki et al., 2008
	• White noise (loud, 71 dB vs. quiet, 51 dB) during touch of rough and fine samples of abrasive paper	Perceived surface roughness	Perceived as smoother (both samples) when paired with the quiet sound (vs. loud and no sound) Perceived as rougher (smooth sample) when paired with the loud sound (vs. quiet and no sound)	Suzuki and Gyoba, 2009
	**Arbitrary sounds – altering music softness (slow tempo, low volume, soft instrumentation, smooth transitions), by:**			
	• Comparing two songs by Sunrise Avenue, “Welcome to My Life” (soft) and “I Don’t Dance” (hard)	Perceived towel softness	Perceived towel as softer when paired with the soft song (vs. hard) No difference in perceived softness vs. music and no sound conditions	Imschloss and Kuehnl, 2019
	• Comparing a soft and a hard version of the same song (“Call Me Maybe” by Carly Rae Jepson)	Perceived towel softness	Perceived towel as softer when paired with the soft version of the song	Imschloss and Kuehnl, 2019
	**Sonification – altering frequency of:**			
	• Sonification of wooden surface being touched by finger (‘grainy’ surface via sounds of rice grains falling into bowl)	Surface texture perception (roughness, hardness, coldness, type of material)	No effect on perceived roughness, hardness Surface perceived as warmer during low frequency (vs. medium/high frequency)	Tajadura-Jiménez et al., 2014
	• Sonification of wooden surface being touched by finger (‘smooth’ surface via sound of a gong after stroking a bell)	Surface texture perception (roughness, hardness, coldness, type of material)	No effect on perceived roughness, hardness Surface perceived as more paper-/sandpaper-like with high frequency (vs. low/medium frequency)	Tajadura-Jiménez et al., 2014
	**Temporal incongruence, of**			
	• Sound (auditory keyboard note) and virtual touch pairings when using a haptic device in a virtual environment	Surface texture perception (roughness); forced choice paradigm of two surfaces	↓ likelihood of identifying haptically identical surfaces as being the same roughness during incongruent auditory condition (vs. congruent and no sound) = altered roughness perception.	McGee et al., 2002

**TABLE 4:** Summary of the effects of non-veridical auditory cues on surface contact properties.

	**Auditory Dimension**	**Perceptual Dimension**	**Findings**	**Studies**
**Auditory cue type: Naturalistic (typical)**	**Decreasing intensity (dB), of:**			
	• Sound of finger tapping (paired to actual finger tapping of a real surface and a virtual surface)	Surface hardness	No effect on perceived surface hardness when tapping a real surface ↓perceived surface hardness (felt softer) when tapping a virtual surface (vs. medium and loud tapping sounds)	Furfaro et al., 2015
	• Biting sound (airborne component) while biting into a potato chip	Chip crispness Chip freshness	↓perceived crispness and freshness; chip perceived as softer and staler (vs. veridical sound)	Zampini and Spence, 2004
	• Biting sound while biting into an apple (No sound condition)	Apple hardness Apple crispness	↓ perceived hardness of apple (vs. veridical sound condition) No effects on perceived crispness	Demattè et al., 2014
	**Increasing intensity (dB), of:**			
	• Sound of finger tapping (paired to actual finger tapping of a real surface and a virtual surface)	Surface hardness	No effect on perceived surface hardness (vs. medium tapping sounds) for either surface	Furfaro et al., 2015
	**Amplifying high frequency components, of:**			
	• Biting sound (airborne component) while biting into a potato chip	Chip crispness Chip freshness	Chip perceived as crisper and fresher (vs. veridical sound)	Zampini and Spence, 2004
	**Attenuating high frequency components, of:**			
	• Biting sound (airborne component) while biting into a potato chip	Chip crispness Chip freshness	Chip perceived as softer and staler (vs. veridical sound)	Zampini and Spence, 2004
	• Biting sound while biting into an apple	Apple crispness Apple hardness	↓ perceived crispness of apple (vs. veridical sound condition) No effects on perceived hardness	Demattè et al., 2014
	**Addition of sounds (audio files recorded during finger tapping on various surface types), to:**			
	• Tapping a surface (index finger) using a haptic device	Stiffness of surface impact	↑perceived stiffness of haptic surface impact with sound cues typically associated with tapping harder surfaces, such as a metal plate (vs. sounds associated with tapping softer surfaces, such as Styrofoam)	DiFranco et al., 1997
**Auditory cue type: Non-naturalistic**	**Sonification - increasing auditory ‘stiffness’ (N/m), of:**			
	• Tapping a virtual horizontal bar with a ‘hammer’ (hand-held stylus, virtual-user interface; sound generated by a physically based sound synthesis model of interacting objects)	Contact stiffness (of ‘hammer’ strike on virtual bar)	↑perceived contact stiffness as auditory stiffness (N/m) increases	Avanzini and Crosato, 1997
	**Sounds with semantic meaning (created using principles of sonification):**			
	• Sounds consistent with wood, metal, snow, and gravel were synthesized and paired with walking (custom system also provided haptic feedback consistent with wood, metal, snow, and gravel); compared congruent and incongruent audio-haptic pairings.^∗^	Walking surface type (wood, metal, snow, or gravel)	When audiotactile information was incongruent, auditory cues were dominant for all pairings	Turchet et al., 2010
	• “Crunchy sound” paired with biting into five types of pureed nursing home food (pseudo-chewing sound created from conversion of the EMG signal from the masseter muscles to audio format, with frequency components modulated)^∗^	Food chewiness Food roughness Food hardness	↑ perceived chewiness for five types of pureed food (vs. no sound) ↑ perceived roughness for two types of pureed food (vs. no sound) ↑ perceived hardness for 1 type of pureed food (vs. no sound)	Endo et al., 2016
	• “Crunchy sound” (as above) paired with biting into food of various textures (Three types of food, each with a pureed-like and mince-like version)^∗^	Food chewiness Food roughness Food hardness	↑ perceived chewiness for pureed-like foods for all three types; ↑ perceived chewiness for mince-like food for two of three types (vs. no sound) ↑ perceived hardness and perceived roughness for pureed-like foods for one food type (vs. no sound) ↑ perceived hardness and roughness for mince-like food for two food types (vs. no sound)	Endo et al., 2017

*N/m, Newtons per meter; EMG, electromyography. ^∗^Difficult classification as these studies could also be considered naturalistic cues given that the auditory cues might be considered ‘typical sounds’ (a result of walking or biting into a textured food). Hence the classification is slightly arbitrary.*

### Auditory Cues and Perception of Surface Texture

**FIGURE 4:**
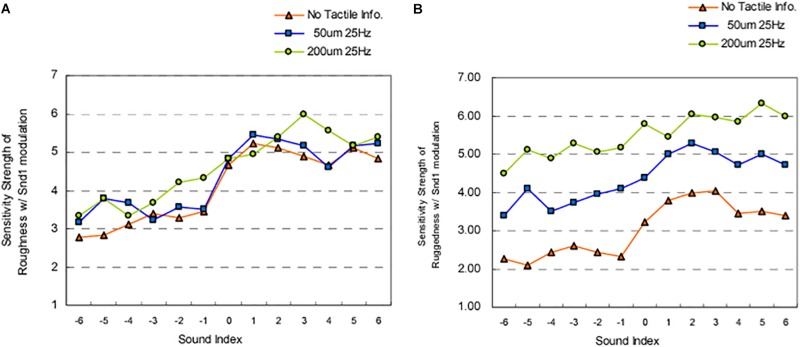
Results from Kim et al.’s (2007) study evaluating the influence of amplifying and attenuating sound frequency components on: **(A)** perceived roughness; **(B)** perceived ‘ruggedness.’ Snd1 = Sound 1 the sound of rubbing sandpaper (Sound 1, grit size #24) was provided alone (“no Tactile info”), or was paired with haptic interaction (index finger) using two different tactile settings on a texture display mouse. The auditory stimuli were divided into various sound frequencies (where 1 = 20–36 Hz; 2 = 36–56 Hz; 3 = 56–96 Hz; 4 = 96 – 174 Hz; 5 = 174 – 284 Hz; 6 = 284 – 584 Hz) that were either amplified by 20 dB (+) or attenuated by 20 dB (–). The sound index (*x*-axis) refers to the frequency-intensity combination. The Sensitivity strength (*y*-axis) refers to ratings provided on 7-point Likert scale (midpoint = neutral) for perceived roughness and ruggedness. These results highlight that amplifying a 30 – 600 Hz frequency range results in increased perceptions of roughness in all conditions. Amplifying a 30 – 300 Hz frequency range results in increased perceptions of ruggedness during virtual haptic interaction (touch conditions) and 56 – 96 Hz during no sound conditions. [From Figures 4, 7 of Kim et al. (2007). Reprinted, with permission from IEEE Proceedings (Computer Society) in the format Republish in a journal/magazine via Copyright Clearance Center].

While manipulating auditory feedback during self-touch, as discussed above, has been shown to alter the perceived properties of one’s own skin, numerous studies have also evaluated the effect of auditory input on the perceived roughness of a surface, using naturalistic auditory feedback of the interaction with the surface. When the auditory feedback is *veridical*, there is no enhancement in the detection of tactile stimuli (vs. visual input alone) (Lederman, 1979) nor enhancement of the ability to discriminate between different abrasive surfaces (Heller, 1982) (although see Ro et al. (2009), for the complexities of such interactions). Further work has demonstrated that when veridical auditory feedback of perceptually salient stimuli (i.e., a rigid probe against a plastic plate) is presented, both tactile and auditory input contribute to the perception of surface texture, but that tactile input tends to be weighted more heavily (Lederman et al., 2002, 2003). However, when naturalistic auditory feedback (i.e., the sound produced by touching a surface) is *non-veridical*, i.e., altered so that the sound provided is not consistent with the sound that one would expect to hear on touching *that* surface, the evidence suggests that auditory cues do matter to tactile contact and the resultant perception of surface qualities. For instance, Guest et al. (2002) demonstrated that amplifying the high frequency sounds of a surface being touched resulted in abrasive sandpaper samples feeling significantly rougher. Meanwhile, attenuating the high-frequency components of the audio signal resulted in an increased perception of surface smoothness as compared to the veridical sound condition (Guest et al., 2002). Such effects on perceptions of surface roughness may be frequency dependent. In contrast to the above findings, Suzuki et al. (2006) showed that attenuating the low- and middle-frequency components of the audio signal resulting from touching abrasive sandpaper samples did not influence perception of surface roughness as compared to a veridical sound condition. However, both sound conditions influenced judgments of surface roughness more than did the absence of sound (Suzuki et al., 2006). Last, there is evidence to suggest that the frequency range that is manipulated has perceptually specific influences for surface texture. Kim et al. (2007) demonstrated that enhancing specific auditory frequencies of the sound of rubbing sandpaper (paired with virtual touch of a haptic device) alters perceived surface roughness and sensations of ‘ruggedness.’ Specifically, amplifying frequencies of 30–300 Hz increase perceived surface ruggedness (Figure 4A) while amplifying frequencies of 30–600 Hz increase perceived surface roughness (Figure 4B). Additionally, the frequency of the sound paired with touch was found to be reciprocally related to the perceived denseness/hardness of the surface – with amplification of sounds (all frequency levels) increasing perceived denseness and attenuation of sounds decreasing perceived denseness (Kim et al., 2007).

Such audio-tactile interactions have been found to extend to influence the perception of surface texture during non-naturalistic sonification of tactile contact and surface exploration. Tajadura-Jiménez et al. (2014) had participants touch and explore a wooden surface with their index finger while real-time sonification of either a ‘grainy’ surface (sounds of rice grains falling into a bowl) or a ‘smooth’ surface (sound of a gong after stroking a steel bell) were provided. The granular synthesis process used altered the sound such that its underlying cause (rice, gong) was no longer identifiable and tactile-to-audio synthesis was used whereby motor behavior shaped the auditory feedback provided. While the frequency manipulation did not significantly alter the perception of roughness for either sound, when high frequency sonification of the gong was used, the material was perceived as more paper-/sandpaper-like than when low or medium frequency sonifications were used instead. Additionally, the surface was perceived as warmer in temperature during the low frequency ‘rice grains’ sonification condition than during the medium or high frequency conditions (Tajadura-Jiménez et al., 2014). That an interaction between sound frequency and temperature should be observed is largely consistent with previous work. People can reliably distinguish between hot and cold water based only on pouring sounds (Velasco et al., 2013a). What is more, the perceived temperature can be manipulated (so that water sounds warmer) by enhancing the volume around 200 Hz and decreasing at 5–6 kHz (and vice versa to perceive temperature as cooler) (Velasco et al., 2013b). Indeed, the experience of drinking warm water is associated with lower-pitched sounds (i.e., lower frequency) while drinking cold water is associated with higher-pitched sounds (Wang and Spence, 2017).

Recent research has shown that audio-tactile interactions influence surface perception even when the surface is virtual. Etzi et al. (2018) evaluated the influence of auditory cues on perceived roughness of haptic virtual surfaces. Haptic surfaces (*n* = 3) were created by varying both the static and dynamic frictional coefficients of a Geomagic Touch device. In brief, touching those surfaces that were paired with the sound of sandpaper (the audio track of a fingertip being rubbed against a piece of sandpaper) were rated as more rough than when combined with the sound of paper (an audio track of a fingertip rubbing a sheet of copy paper) or no sound (Etzi et al., 2018). Additionally, one of the surfaces that was presented (a very smooth tactile surface) was rated as less rough (i.e., smoother) when paired with the paper sound than when no sound was presented.

According to the evidence that has been published to date, non-naturalistic, arbitrary auditory cues, in the form of white noise, can influence the perception of touched surfaces. White noise (62 dB) paired with the touching of abrasive samples was found to alter the perception of roughness of courser surfaces (grit values of 1200 and 4000), but not of finer surfaces (grit values of 400 and 600) (Suzuki and Gyoba, 2007). In addition, touching stimuli in synchrony with a change in loudness of the white noise (four levels of loudness changed in a pseudorandom order) was found to influence estimates of tactile roughness regardless of the direction of the change, but not tactile estimates of length (Suzuki et al., 2008). Specifically, participants touched abrasive paper of 14 different particle sizes and of 14 different lengths and rated their subjective feelings of roughness and of length. Auditory stimuli were paired with index and middle finger touch of the paper; the white noise intensity changed at 1 s intervals (control stimulus 5 beeps, 1000 Hz, 64 dB), with a change in the direction of touch paired with intensity changes. Changing the loudness of the white noise decreased the slope of the roughness estimation function (vs. control) (Suzuki et al., 2008) – i.e., smaller differences in the perception of roughness, despite actual differences in particle size of the paper. By contrast, pairing the tactile contact of the abrasive papers with pure tones (1000 Hz) had no effect on either the estimation of roughness or of length, thus suggesting that it is not merely the presence of sound by itself that influences perception (Suzuki et al., 2008). Similarly, the loudness of white noise (vs. change in loudness tested above) also appears important to the perception of surface roughness (Suzuki and Gyoba, 2009). Synchronizing quiet vs. loud sounds (51 vs. 71 dB) with movement in which abrasive paper is touched (with the index finger moving back–and-forth) revealed that when quiet sound (congruent with touching a finer texture) was presented, the rough and the fine samples were judged to be smoother than when the loud sound or else no sound was presented (Suzuki and Gyoba, 2009). By contrast, the loud sound (congruent with stroking a coarse texture) resulted in the smooth sample being perceived as rougher (Suzuki and Gyoba, 2009).

Providing non-naturalistic auditory textural cues can influence the perception of roughness for virtual textures. Specifically, using the PHANToM^TM^ 1.0 force feedback device (SensAble), McGee et al. (2002) were able to generate virtual textures with participants interacting with the virtual texture by means of a pen-like stylus (passively supported in the hand). The participants moved the stylus back and forth across the virtual texture and this was either paired with congruent sound (single MIDI note generated from the peak of every rise of the virtual texture), incongruent sound (auditory frequency 120% of the haptic frequency – i.e., the number of auditory tones was 20% higher than the number of haptic bumps), or else no sound was presented. A forced choice paradigm was used in which the participants compared the perceived roughness of any two virtual textures. The likelihood that haptically identical textures were perceived as the same roughness was significantly lower in the multisensory conditions than in the haptic only (i.e., no sound) condition. When the haptic and auditory stimuli were *incongruent*, the likelihood that haptically identical textures were perceived as the same roughness was significantly lower than when the haptic and auditory stimuli were *congruent* (McGee et al., 2002). Such findings suggest that non-veridical auditory input can provide important cues as far as the perception of surface roughness, even in virtual environments. Other research has highlighted the complexity of roughness perception as far as virtual surfaces are concerned, showing improved virtual texture discrimination with two and three-modality (visual, auditory, and haptic) conditions but only for certain combinations of stimuli and not others (Weisenberger and Poling, 2004).

Most recently, the influence of non-naturalistic, arbitrary auditory cues on perceived surface ‘softness,’ in the context of the retail environment, has been explored. In the study by Imschloss and Kuehnl (2019), participants touched a towel and rated its perceived haptic softness when ‘soft’ vs. ‘hard’ music was paired with the tactile interaction. ‘Soft’ music was pre-tested and identified as that with slow tempo, low volume, and harmonic legato-like sounds (soft instrumentation with smooth transitions). In brief, a towel was perceived as softer when paired with a ‘soft’ (vs. a ‘hard’) song, and this finding held when two versions (‘soft’ and ‘hard’) of the same song were used (i.e., haptic influences not due to song lyric differences). Interestingly, the influence of audio ‘softness’ on perceived towel softness only occurred when touching a soft fabric (did not alter perceived surface texture properties of a ‘non-soft’ fabric), and only when people were unaware (vs. aware) that music can influence their perceptions of textiles. Last, environment features appear important to such an effect: haptic softness perceptions were only modulated by soft music when consumers were standing on a hard (vs. soft, carpeted) flooring. Interestingly, similar effects of ‘soft’ music have been seen in gustation: chocolate is perceived as creamier and sweeter when paired with ‘soft’ (vs. ‘hard’) music (Reinoso Carvalho et al., 2017).

### Interim Summary

The evidence that has been published to date shows that auditory cues clearly contribute to the multisensory perception of surface texture during self-generated touch. That both naturalistic auditory cues and non-naturalistic auditory cues influence perceived surface roughness provide support for the influence of auditory cues at a perceptual level (i.e., as a result of multisensory integration) rather than solely at a cognitive, decisional level (e.g., this sounds rougher therefore it must be rougher).

**FIGURE 5:**
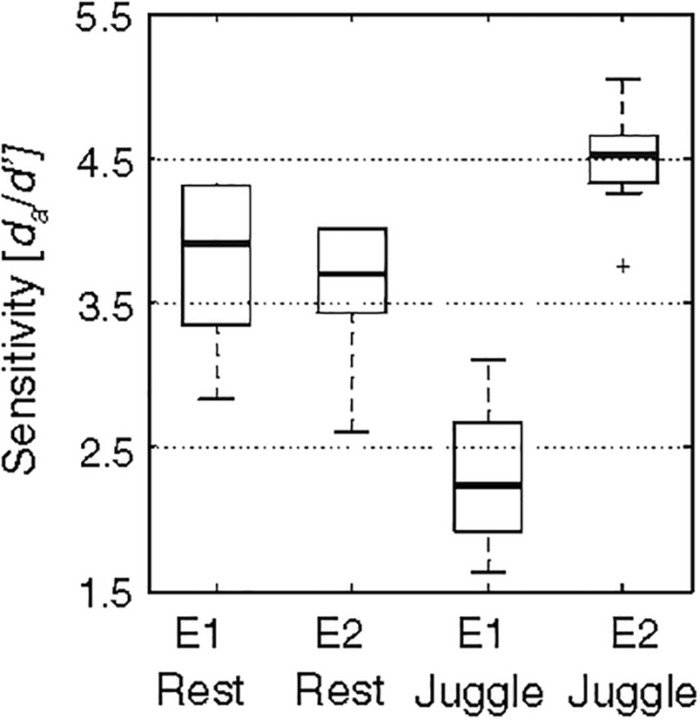
Results from Juravle and Spence’s (2011) study highlighting that movement-induced sensory perception changes are specific to the type of sensory input and its relevance to the movement. E1 refers to Experiment 1 and shows that the sensitivity (*y*-axis) in detecting a gap in *tactile* stimulation was lower when delivered to the hand that was juggling than when delivered to the hand at rest. E2 refers to Experiment 2 and shows that the sensitivity in detecting a gap in *auditory* stimuli was higher when delivered during the juggling condition. Box plots represent the middle 50% of data (dark line = median) and whiskers represent the interquartile range (+sign = values 1.5 times the inter-quartiles range). [From Figure 1a of Juravle and Spence (2011). Reproduced with permission of Springer Nature under Copyright Transfer Agreement and License to Publish Agreement of the author which allows adaption of figures for style and formatting purposes under the condition that this does not alter the meaning of the content].

Given the interactive possibilities of altering perceived surface texture via sound [for use in VR shopping applications, Ho et al. (2013) for example], the bi-directional relationship between sensory perception and movement is relevant to consider. Recent work shows that changes in sensory perception during movement are specific to the type of sensory input and its relevance to the movement concerned (Juravle and Spence, 2011; Juravle et al., 2017). For example, when auditory cues are paired with a juggling task, there is an increased sensitivity to detect a gap in the auditory cue when compared with a rest (no movement) condition, but the opposite occurs for tactile cues: reduced sensitivity occurs during the movement (juggling) condition than during rest (see Figure 5) (Juravle and Spence, 2011). Such differential effects are proposed in line with forward models of motor control (Wolpert et al., 1995; Miall and Wolpert, 1996) whereby the movement of our limbs is thought to result in the suppression of the information related to the movement itself (e.g., tactile information), but enhanced perception of external stimuli, such as auditory information. Given this, in some situations, auditory cues may shape perception of surface contact features to a greater extent than tactile input, because we may be more sensitive to any changes in the auditory cues.

However, it also appears that the sensory input needed to successfully perform a goal-directed task is important in determining the occurrence of sensory suppression. Visually guided goal-directed movement (i.e., a reaching task to visual targets) suppresses auditory perception (Manson et al., 2018) – that is, we hear less when we are actively moving. Such suppression has been posited to be due to context-dependent sensory re-weighting (Manson et al., 2018) whereby task-irrelevant sensory information (e.g., audition for a visually guided task) is suppressed via sensory gating (Chapman et al., 1987). These findings suggest that when high levels of performance are needed for a task, sensory cues would need to be task-relevant or else their effect may be minimal to non-existent, given such suppression. An extension of this hypothesis would then suggest that when high levels of performance are needed for a task in which auditory information is crucial, the latter should no longer be suppressed, but visual or tactile input might be. Such findings may be relevant for a number of VR applications, such as clothes shopping, given that we would rely less on vision, and in the absence of tactile input, auditory input may be highly relevant to the task of selecting which item of clothing to purchase. Even in the presence of tactile input, auditory input may be relevant: pairing the sound of fabric to an AR retail clothing application has also been shown to increase immersion and interaction with the application (Ho et al., 2013). Tactile-auditory pairings (particularly if non-veridical and surprising) provide unique experiences: such interactions are being explored by artists who are working at the intersection of touch and sound (Bourbonne, 2018) and those working in VR to generate illusions of material compliance of a virtual object using a VR hand-held controller (Lee et al., 2019).

### Auditory Cues and Perception of Surface Contact Properties

Auditory cues have also been explored in terms of their ability to alter perception of the surface contact properties, such as surface stiffness, surface hardness, or the perception of the type of material being interacted with. Table 4 provides a summary of studies that have evaluated the influence of auditory cues on surface contact properties.

Auditory cues have been shown to influence the perception of contact stiffness in virtual surfaces (Avanzini and Crosato, 1997; DiFranco et al., 1997). In a study by Avanzini and Crosato (1997), participants used a Phantom Omni device^TM^ (virtual-user interface) – a hand-held stylus – and then judged the stiffness of the impact between the “hammer” (represented by the stylus) and a horizontal gray bar represented using a 2D virtual display on a computer monitor (Avanzini and Crosato, 1997). The ‘stiffness’ of the auditory input was manipulated (by altering auditory correlates of impact force), which resulted in auditory stiffness levels falling somewhere between those of ‘wood’ and ‘glass.’ The participant’s estimations of contact stiffness were significantly altered based on auditory input, with increases in rated contact stiffness occurring as levels of auditory stiffness increased, this despite a lack of any change in haptic stiffness provided by the device (Avanzini and Crosato, 1997). Such findings have been supported by DiFranco et al. (1997) who found that when impact sounds were paired with tapping a surface (using a haptic device), the perceived surface impact was judged as stiffer when non-veridical sound cues typically associated with tapping harder surfaces were presented. Interestingly, naïve subjects (i.e., those with no experience of using the Phantom) were more affected by these sound cues than were subjects who had experience using the Phantom (DiFranco et al., 1997).

In contrast, pairing non-veridical auditory feedback of tapping sounds (quiet, medium, and loud) with finger tapping, did not alter perceptions of surface hardness when real surfaces were tapped, but it did in the case of virtual surfaces (Furfaro et al., 2015). When tapping the virtual surface (i.e., no tactile cues available), participants perceived the tapped surface as softer when tapping was paired with the quiet sound as compared to the medium and loud tapping sounds (Furfaro et al., 2015). Such effects suggest that ambiguity, or lack of feedback, may increase the ability of auditory cues to shape perception. Indeed, an additional study provided support that modulation of perceived surface features (via auditory cues of tapping) occurs without the need for tactile or proprioceptive input (Fujisaki et al., 2014). That is, even when a person is merely viewing a video of a hand tapping (without self-movement), perceptions of surface type (e.g., wood, metal, ceramic, glass) are altered by non-veridical auditory cues (Fujisaki et al., 2014).

Influences of auditory cues on perception of contact surfaces extend to those experienced while walking (Turchet et al., 2010). Using specialized sandals with embedded pressure sensors and actuators, Turchet et al. (2010) used a customized system to provide real-time, synthesized auditory and haptic sensations of walking on difference surfaces. Previous work has confirmed that participants could delineate these different surfaces based on auditory and haptic cues. The customized system specifically mimicked hard surfaces (wood and metal) and aggregate surfaces (snow and gravel). When incongruent input was provided (auditory: wood; haptic: snow), auditory stimuli were dominant and shaped perception of the surface being walked on.

**FIGURE 6:**
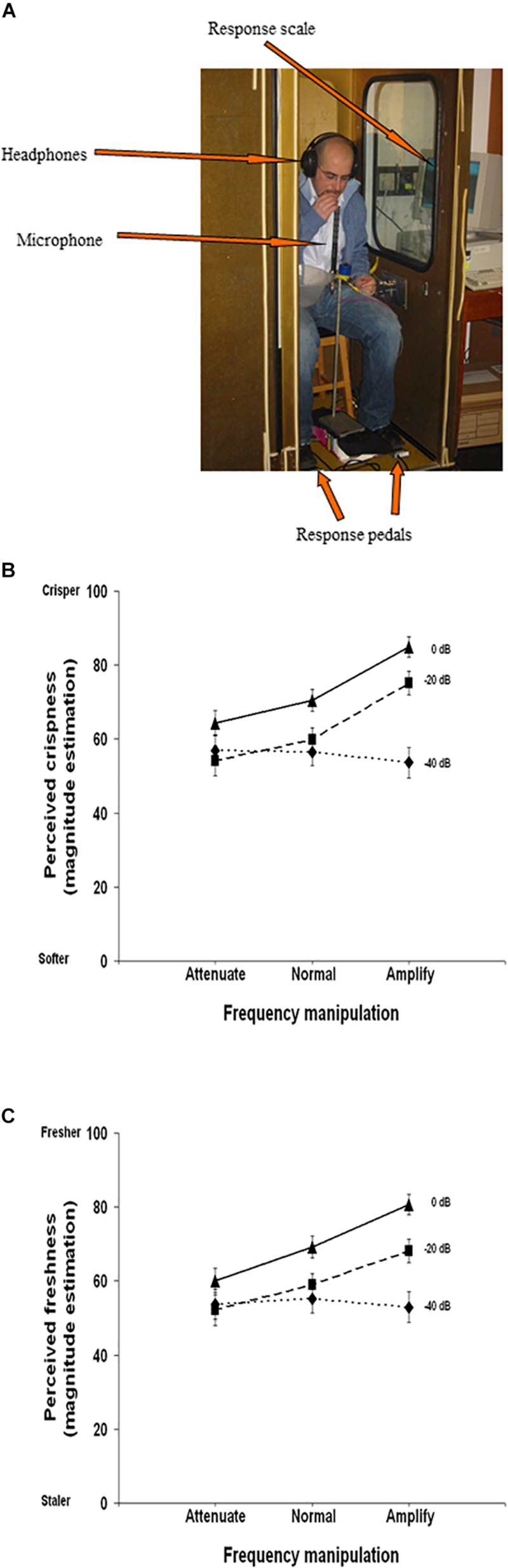
Experimental set-up and results from Zampini and Spence’s (2004) study demonstrating the influence of manipulating biting sounds (airborne component) when biting into a potato chip. **(A)** Experimental set-up of participant; note that during testing, the booth door was closed and participants provided responses via computer screens situated through the wall (left-hand side) of the booth. **(B)** Perceived crispness of the chip (*y*-axis) during sound frequency alteration (*x*-axis) and 3 sound intensity conditions (0 dB, –20 dB, –40 dB). Results show that amplifying the high frequency components of the sound increased perceptions of crispness (unless sound is very quiet: –40 dB), and decreasing sound intensity increase perceptions of chip softness. **(C)** Perceived freshness of the chip (*y*-axis) during sound frequency alteration (*x*-axis) and 3 sound intensity conditions (0 dB, –20 dB, –40 dB). Amplifying the high frequency components of the sound increased perceptions of freshness (unless sound is very quiet: –40 dB), and decreasing sound intensity increase perceptions of chip staleness. [From Figures 1, 2a,b of Zampini and Spence (2004). Reproduced with permission of John Wiley & Sons publication under author agreements which allow the author reuse of up to three figures to republish in a new journal article].

Finally, auditory cues can impact our perception of the tactile contact induced by biting or chewing while eating. Modifying the audio feedback while biting into a potato chip (namely the airborne component of the biting sound – as opposed to the bone-conducted sounds) changes people’s perception of the texture of the food that they are eating (Zampini and Spence, 2004). Increasing the overall sound level and/or amplifying just the high frequency components of the biting sound resulted in the potato chips being rated as fresher and crisper. By contrast, reducing the sound, and attenuating the high frequency components resulted in the potato chips being judged as both softer and staler (see Figure 6). Similarly, attenuating the high frequency components of the sound of biting into an apple resulted in the apple being judged as less crisp than during a veridical sound condition, and globally reducing sound input (microphone turned off) resulted in reductions in perceived hardness of the apple (vs. veridical sound condition) (Demattè et al., 2014). When non-veridical auditory feedback of chewing is provided, perception of food texture is also altered (Endo et al., 2016). Specifically, Endo et al. (2016) used the signal from the electromyogram (EMG) of participant’s masseter muscle and converted it to an audible format to create a pseudo-chewing sound. This pseudo-chewing sound was then manipulated by modifying the frequency properties (i.e., in order to create a “crunchy sound”). When the “crunchy sound” was paired with biting, the perceived ‘chewiness’ of the pureed (i.e., without texture/sonic interest) nursing home food was greater than during the no-sound condition. This increase in perceived chewiness occurred for four of the five different kinds of pureed nursing care foods. Additionally, the perceived roughness (on a smooth-rough scale) of food textures was significantly greater (in two of the foods), and perceived hardness (in one of the foods) was significantly enhanced in the EMG sound condition as compared to the no-sound condition. Such impacts of auditory cues (i.e., the altered sound of the EMG output) on perceived food texture have been shown to extend to pureed foods, although the perceptual effects are not as large as when the food is texturally inhomogeneous (i.e., minced food) (Endo et al., 2017).

### Interim Summary

The evidence reviewed in this section suggests that auditory influences on perception extend to numerous forms of self-generated movement – touching a surface, walking, and chewing. While various studies have shown large influences on perception of manipulating naturalistic sounds (DiFranco et al., 1997; Zampini and Spence, 2004; Demattè et al., 2014; Furfaro et al., 2015), use of non-naturalistic auditory cues, as seen in the creation of a pseudo-chewing sound also appear to hold promise. Indeed, a recent system of embodied sound interaction – termed Gamelunch – used a sonic feedback system to provide contradictory auditory input when people performed actions such as cutting, piercing, pouring, grasping, stirring, and mixing (Delle Monache et al., 2008). For example, when using salad spoons to mix the salad while dressing it, a crumpling sound was sonified such that a dense granular sound (like that of sand) was paired with the mixing motion (Delle Monache et al., 2008). This auditory change was reported to result in a sensation of interacting with a heavier material (Delle Monache et al., 2008) and in some contradictory sound and object interaction/movement pairing, resulted in participants actively seeking out new ways to move the object. Such findings suggest that compelling changes in surface (and movement) perception can occur, but also, that when auditory cues are temporally paired with actual movement, even entirely contradictory auditory cues can be embodied. Continual improvement in engineering new sounds is occurring: work by Klatzky et al. (2000) shows that altering damping (how sounds decay over time) influences perception of object contact properties, including perceived length of a bar hitting a surface and classification into categories of glass, rubber, wood, and steel. Additionally, Zhang et al. (2017) recently put forward a computational learning system to infer properties of a falling object based only on sound. The model learns to map sound waves to object properties and then uses predicted values to guide the inference (approximation of human priors based on past experience). This system had similar (or better) classification accuracy than humans at identifying objects (such as ‘with edge’ or ‘with curved surface). There is also work exploring the optimal auditory rendering during walking in simulated environments (Serafin et al., 2012).

That perceptual alterations induced by auditory cues are enhanced in situations of higher sensory ambiguity (i.e., VR where visual input may not be fully accurate via 2D presentation or temporal delay) as found here supports the theory of MLE cue integration (Ernst and Banks, 2002). Indeed, if sensory input is noisy or ambiguous, a reliable input (the naturalistic sound of movement) is weighted more heavily (Ernst and Banks, 2002) and thus, has a greater opportunity to shift people’s perception. Such knowledge may have implications for the use of movement sonification to improve motor learning and performance (Effenberg et al., 2016), namely taking advantage of an enhanced ability to adapt motor performance based on auditory cues when using VR in athletic training or rehabilitation.

Relevant extensions of audiotactile interactions occur for the neurological field. For example, alloesthesia is rare clinical condition that involves a disorder of spatial localization. Following brain injury, patients experience a given stimuli provided on the contralesional side of the body opposite to the side of actual stimulation (e.g., touched on the left, yet perceive it on the right). In a case study published in 2005, Ortigue et al. (2005) demonstrated the existence of multisensory, audiotactile alloesthesia. Irrelevant stimuli (both tactile and auditory) induced a mislocalization of tactile or auditory stimuli of the other modality (always mislocalized from the contralesional left side to specific body areas of the right side). Tactile and auditory pairs provided on the same side of the body were accurately identified (control) and similar competition did not occur for other sensory pairings (e.g., vision). That a systematic distortion of tactile localizations from the left-sided lower body parts to the right side of the face occurred, suggests precise anatomical and spatial rules of mislocalization. Thus, exploring auditory influences on touch may have important contributions to the understanding of rare and complex clinical conditions.

## Influence of Auditory Cues on Emotional Response and on the Pleasantness of Movement-Related Activity

Moving, or interacting, with the environment can give rise to strong emotional responses. For example, just think of the relieving feeling of moving after sitting in a cramped seat, or the vexing feeling of squeezing through a tight passageway. Certain movements are also more pleasurable than others, and this pleasure is likely influenced by multisensory cues: a walk in the sunshine is much more pleasant than a walk through a loud and busy airport, say. Auditory cues during movement thus have the potential to impact on both the emotional response induced by the movement and on the overall pleasantness of the multisensory experience. Furthermore, it is known that sounds have clear and varied emotional valences, as illustrated by Bradley and Lang’s seminal work (Bradley and Lang, 2000) characterizing the range of affective valences that are associated with sound (unpleasant vs. pleasant). Moreover, recent work from Fritz et al. (2017) highlights that the emotional valence of sound can have multimodal influences, namely, that the perceived sexiness of music influences subjective ratings of the sexiness of gentle touch stimulation. Together, these findings suggest that there is scope for auditory cues, when paired with movement, to influence the emotional response that may be elicited. Table 5 presents a summary of the findings in this section.

**TABLE 5:** Summary of the effects of non-veridical auditory cues on emotional response and on the pleasantness of movement-related activity.

	**Auditory Dimension**	**Perceptual Dimension**	**Findings**	**Studies**
**Auditory cue type: Naturalistic (typical)**	**Addition of auditory cues to:**			
	• Touch of haptic surface (various virtual surfaces varying in roughness created via Geomagic Touch device; used sound of a fingertip rubbing against sandpaper and sound of a fingertip rubbing against a sheet of copy paper)	Valence (pleasantness)	↓pleasantness of all surfaces when touch combined with ‘sandpaper sound’ (vs. ‘copy paper sound’ and no sound condition) ↓pleasantness during audio-tactile incongruence, e.g., a very smooth tactile surface when paired with ‘copy paper sound’ (vs. no sound)	Etzi et al., 2018
	**Altering intensity (dB), of:**			
	• Sound of high heels while walking (via combinations of surface type [ceramic, carpet] and sole type [leather, polypropylene])	Valence (pleasantness) Arousal (intensity of emotional response evoked) Dominance (degree of control experienced) Bodily feelings	↑pleasantness when hearing sounds generated by ceramic floors (vs. carpet) ↑ arousal during louder sounds (ceramic/polypropylene > ceramic/leather; ceramic/polypropylene > carpet/polypropylene; ceramic/leather > leather/carpet) ↑ perceived dominance when hearing sounds generated by ceramic floors (any sole) than carpet; ↑ dominance for ceramic/leather > both ceramic/polypropylene and carpet/leather) ↑ ‘contented’ feeling with sound of contact with ceramic (any sole) > contact with carpet (any sole)	Tonetto et al., 2014
	• Sound of finger tapping (paired to actual finger tapping of a real surface and a virtual surface)	Valence (pleasantness) Arousal (galvanic skin response)	↓pleasantness when tapping a real surface paired with quiet sound (vs. loud sound) – i.e., during audio-motor incongruence ↑ arousal when tapping a virtual surface paired with quiet (vs. medium or loud condition)	Furfaro et al., 2015
	**Amplifying high frequency components, of:**			
	• Walking auditory feedback	Valence (pleasantness) Arousal (galvanic skin response)	↑ pleasantness rated during high frequency amplified footstep sounds (vs. low frequency and no sound condition) ↑arousal during high frequency amplified footstep sounds (vs. low frequency and no sound condition)	Tajadura-Jiménez et al., 2015a
**Auditory cue type: Non-naturalistic**	**Sounds with semantic meaning (created using principles of sonification):**			
	• “Crunchy sound” paired with biting into five types of pureed nursing home food (pseudo-chewing sound created from conversion of the EMG signal from the masseter muscles to audio format, with frequency components modulated)^∗^	Pleasantness Satisfaction Enjoyment/excitement Engagement in an actual eating experience	↑ pleasantness, satisfaction, enjoyment/excitement, and a feeling of engagement in an actual eating experience when eating pureed nursing home foods in the condition with sound added (vs. no sound)	Endo et al., 2016
	• “Crunchy sound” (as above) paired with biting into food of various textures (three types of food, each with a pureed-like and mince-like version)^∗^	Pleasantness Satisfaction Enjoyment/excitement Engagement in an actual eating experience	Generally, ↑ pleasantness, satisfaction, enjoyment/excitement, and a feeling of engagement in an actual eating experience when eating for both pureed-like and mince-like foods in the condition with sound added (vs. no sound)	Endo et al., 2017

*^∗^Difficult classification as these studies could also be considered naturalistic cues given that the auditory cues might be considered ‘typical sounds’ (a result of walking or biting into a textured food). Hence the classification is slightly arbitrary.*

There is evidence to suggest that the non-veridical, naturalistic auditory cues provided during touch influence the perceived pleasantness of the interaction. For instance, the influence of such auditory cues on the pleasantness of haptic virtual surfaces was recently evaluated (Etzi et al., 2018). As discussed in the surface texture section above, haptic surfaces with various textures were created via a Geomagic Touch device. In brief, touching those surfaces that were paired with the sound of sandpaper (the audio track of a fingertip being rubbed against a piece of sandpaper) were rated as less pleasant than when combined with the sound of paper (an audio track of a fingertip rubbing a sheet of copy paper) or no sound (Etzi et al., 2018). There was some evidence to suggest that incongruence in terms of audio-tactile cues may also influence ratings of pleasantness: one of the surfaces that was presented (a very smooth tactile surface) was rated as less pleasant when paired with the paper sound than when no sound was presented. This was hypothesized to be attributable to a disconnect between touch and auditory cues in the former condition (Etzi et al., 2018). Similarly, the non-veridical interactive audio-feedback of the sound of a finger tapping influenced both the pleasantness (valence) and arousal ratings of the tapping motion. Specifically, the experience of tapping a real surface was rated as significantly less pleasant when paired with a quiet (vs. loud) tapping sound. While altering the auditory cues paired with tapping on a virtual surface failed to influence pleasantness ratings, arousal ratings were altered: a quiet tapping sound increasing arousal ratings (as compared to a medium or loud sound condition) (Furfaro et al., 2015). These results therefore suggest that audio-motor incongruence (i.e., audio feedback of the quiet sound does not match expectations based on applied strength) can give rise to experiences that are both unpleasant and arousing.

Such findings are supported by work that provided non-veridical, but naturalistic, auditory feedback while women were instructed to walk in high heels (Tonetto et al., 2014). Tonetto et al. (2014) found that manipulating the auditory feedback of the sound of high heels while participants were walking, including both the type of high heel sole (leather vs. polypropylene) and the type of flooring (ceramic or carpet), altered emotional outcomes. In particular, louder sounds resulted in higher pleasantness (affective valence), arousal (intensity of the emotional response evoked), and dominance (the degree of control experienced by the person) ratings. The sound of contact with ceramic surfaces (with any sole) resulted in higher ‘contented’ scores than those involving contact with carpet and audio feedback associated with a polypropylene sole on carpet generated higher ratings for feelings of ‘softly relaxed,’ ‘at ease,’ ‘contented,’ and ‘resentful.’ In contrast, audio feedback of the sound of a leather sole on a ceramic floor generated higher scores for ‘comfortable’ and ‘contented’ than the sound of a leather sole on carpet and higher scores for ‘at ease’ and ‘contented’ than the sound of a polypropylene sole on ceramic flooring. Similarly, Tajadura-Jiménez et al. (2015a) demonstrated that when the sound produced by foot-steps was altered, with high frequency components amplified (which, as noted above, resulted in participants feeling lighter), participants also reported the experience to be more pleasant than the condition where low frequency components were amplified. Additionally, arousal (as measured via GSR) was increased when the high frequency components of walking sounds were amplified as compared with the low frequency and no sound conditions (Tajadura-Jiménez et al., 2015a).

Last, providing non-veridical auditory feedback of chewing sounds has been shown to alter the perceived pleasantness of the food being eaten (Endo et al., 2016). Specifically, pairing a “crunchy sound” with chewing significantly enhances ratings of pleasantness for the pureed nursing care foods (vs. no-sound condition) (Endo et al., 2016), regardless of the texture of the food (Endo et al., 2017). And while pleasantness was not directly measured, findings that show that modifying audio feedback of biting into a potato chip or an apple alters perceptions of ‘crispness’ (Zampini and Spence, 2004; Demattè et al., 2014) are relevant to consider given previous work that demonstrates that sounds denoting ‘crispness’ are rated as the most pleasant (Vickers, 1983; Spence, 2015).

### Interim Summary

The studies reviewed in this section suggest that auditory cues can have a marked impact on emotional responses during movement. The results highlight that perceived incongruence between the movement-relevant information contained within the auditory cue and the motor action itself appears to play a role in the rated pleasantness of the experience. That is, even when the sensory inputs are temporally congruent, differences between the expected loudness of the auditory input given the force applied during the movement and the actual loudness of auditory input influence pleasantness. Such effects suggest that the expectations of movement outcome include sound, a finding that is supported by neuroimaging results showing that there are similar neural substrates for both movement and the sound it produces (Gazzola et al., 2006). Moreover, that auditory cues play a role in perceived gustatory pleasantness highlights the relatively under-valued importance of what we hear when we eat (Spence, 2015). Emotional responses can be induced even by sounds that do not have typical meaning – that is, when merely tone and noise features of auditory input are altered (Vastfjall, 2012). Given this, it is relevant to consider, and to further explore, the notion of bringing sonification to eating and drinking.

## Influence of Auditory Cues on Movement and Behavior

When we move, predictions of the outcome of movement are made and then evaluated in terms of their congruence with the actual movement. In particular, an efference copy of the motor action is created and then compared to the sensory feedback derived from the movement that has been executed (Wolpert et al., 1995). If the movement was not executed as planned, then movement patterns are updated. Importantly, the sensory feedback is multisensory – for example, reaching to touch a seen object will provide visual, proprioceptive, tactile, and potentially also auditory cues. As such, auditory cues that provide non-veridical feedback of a movement outcome may be used to assess the impact of such cues on movement and/or behavior. This section explores those studies that have used non-veridical naturalistic and non-naturalistic sound (including sonification) in order to influence changes in movement pattern or in behavior. A summary of the findings of this section are presented in Table 6.

**TABLE 6:** Summary of the effects of non-veridical auditory cues on motor behavior.

	**Auditory Dimension**	**Movement Dimension**	**Findings**	**Studies**
**Auditory cue type: Naturalistic (typical)**	**Addition of auditory cues to:**			
	• Reach and grasp of various objects with surfaces of aluminum, paper, string, wool (used recorded sounds of grasping object covered in the same materials)	Movement duration Deceleration time Grasp closing time Contact behavior (of grasp when stimulus object had differing material on upper and lower half)	↑movement duration, deceleration time, and grasp closing time when sounds were incongruent with surface grasped (vs. congruent object and sound) Auditory cue guided contact behavior (if sound of paper, participants used sound-congruent grasp, i.e., chose to grasp paper half of object)	Castiello et al., 2010
	• Touch of haptic surface (various virtual surfaces varying in roughness created via Geomagic Touch device; used sound of a fingertip rubbing against sandpaper and sound of a fingertip rubbing against a sheet of copy paper)	Exploration time of virtual surface	No effect of sound on surface exploration time (or sound × virtual surface interaction)	Etzi et al., 2018
	**Altering intensity (dB), of:**			
	• Sound of finger tapping (paired to actual finger tapping of a real surface and a virtual surface)	Maximum finger acceleration Changes in finger acceleration over time Speed of tapping	↑ maximum acceleration values when tapping paired with quiet sound (vs. loud sound; both surfaces) ↑change in acceleration over time when tapping a real surface paired with quiet sound (vs. medium and loud sound) and largest individual variation ↑ speed of tapping and ↓ maximal acceleration (both surfaces) when any audio feedback (low, medium, and high) paired with tapping (vs. no sound)	Furfaro et al., 2015
	**Altering frequency components, of:**			
	• Walking auditory feedback	Heel contact time Upward foot movement acceleration	↑ heel contact time with ground in low frequency condition (vs. high frequency condition) ↑upward foot movement acceleration in the high frequency condition (vs. low frequency condition)	Tajadura-Jiménez et al., 2015a
	**Spatial incongruence (vs. actual location), of:**			
	• Sound of finger tapping on table. *Temporally congruent* (synchronous) finger tapping/sound of finger tapping resulted in a feeling of having an elongated arm. *Temporally incongruent* (Asynchronous) finger tapping/sound of tapping did not result in changes to the perceived size of the arm.	During a goal-directed reach task following auditory adaptation: Mean velocity Peak velocity Peak acceleration Latency peak velocity Latency peak acceleration Reached position Movement time	After synchronous finger tapping (inducing feeling of an elongated arm): ↓mean velocity when reaching (vs. asynchronous) ↓ peak velocity when reaching (vs. asynchronous) No effects on peak acceleration or latencies No effect on reached position ↑movement time when reaching (vs. asynchronous) driven by those with a morphologically long arm (vs. short arm)	Tajadura-Jiménez et al., 2016
	**Altering congruence of visuo-auditory cues [based on past/training experience], of:**			
	• Piano musical notes in pianists (visually providing a note to play [e.g., C], and temporally pairing with congruent [C] or incongruent [D] auditory feedback)	Response time (to play piano key) Error	↑Response time (slower) in incongruent condition than congruent ↑errors in incongruent condition No effects seen in non-pianists	Drost et al., 2005
	**Altering temporal congruence, of:**			
	• Walking auditory feedback	Walking speed after stride (foot lift off to next step) Walking speed at each stride (during foot contact)	↑ speed of foot lift off to next step with increasing auditory delay of sounds of footsteps (vs. congruent) ↓speed at time of stride (during foot contact) with increasing auditory delay of sounds of footsteps (vs. congruent)	Menzer et al., 2010
	• Breathing auditory feedback (natural breath sounds), with post-expiration pause manipulated	Breath pattern	Participants were more likely to match their breath patterns to non-veridical natural breath sounds (vs. sonified versions of breath sounds [see below] or a no-sound control condition)	Murgia et al., 2016
**Auditory cue type: Non-naturalistic**	**Sounds with semantic meaning (sound created using principles of sonification):**			
	• “Crunchy sound” paired with biting into five types of pureed nursing home food (pseudo-chewing sound created from conversion of the EMG signal from the masseter muscles to audio format, with frequency components modulated)^∗^	Mastication intensity (normalized root mean square of EMG signal) Mastication rhythm (Hz)	No effect of sound (vs. no sound) on either mastication intensity or rhythm	Endo et al., 2016
	• “Crunchy sound” (as above) paired with biting into food of various textures (three types of food, each with a pureed-like and mince-like version)^∗^	Mastication intensity (normalized root mean square of EMG signal) Mastication rhythm (Hz)	No effect of sound (vs. no sound) on either mastication intensity or rhythm	Endo et al., 2017
	• Sounds depicting snow and mud were temporally paired with walking in different emotional styles (also used naturalistic recorded sounds of walking on linoleum and on a wood floor)^∗^	Heel to toe Inter-Onset-Interval (IOI, measured in ms; for left foot and for right foot) Heel to heel IOI (ms: between left and right foot)	No overall effect of sound on heel-to-toe IOI (neither right nor left foot) No overall effect of sound on heel-to-heel IOI “Tendency” for: ↑both heel-to-toe and heel-to-heel IOI (walk faster) when sounds depicting snow were paired to walking ↓ both heel-to-toe and heel-to-heel IOI (walk slower) when sound depicting mud were paired to walking	Bresin et al., 2010
	**Sonification – altering frequency of:**			
	• Sonification of wooden surface being touched by finger (‘grainy’ surface via sounds of rice grains falling into bowl)	Applied finger pressure Velocity of movement	↓finger pressure when sound was low frequency (vs. medium or high frequency) No effect on movement velocity	Tajadura-Jiménez et al., 2014
	• Sonification of wooden surface being touched by finger (‘smooth’ surface via sound of a gong after stroking a bell)	Applied finger pressure Velocity of movement	No effect on finger pressure ↓ speed of movement when sound was medium frequency (vs. low or high frequency)	Tajadura-Jiménez et al., 2014
	**Sonification – altering temporal congruence, of:**			
	• Sonified breathing auditory feedback (with rising tones for inspiration and falling tones for expiration), with post-expiration pause manipulated	Breath pattern	No more likely to alter their breathing pattern to match incongruent sonified breath sounds than in a no-sound control condition (which depicts natural breathing pattern alteration)	Murgia et al., 2016

*^∗^Difficult classification as these studies could also be considered naturalistic cues given that the auditory cues might be considered ‘typical sounds’ (a result of walking or biting into a textured food). Hence the classification is slightly arbitrary.*

Previously, it has been shown that when auditory cues are *veridical*, motor performance can be improved, relative to no-sound conditions. For example, in an audio-visual interface (the Ballancer^TM^), non-naturalistic auditory cues (consisting of sonic feedback of a rolling ball or of abstract feedback) that provide veridical velocity information improve performance of moving a ball to a target area (Rath and Rocchesso, 2005; Rath and Schleicher, 2008). Importantly however, recent research suggests that such effects on motor performance also occur when auditory cues are *non-veridical*. Providing real-time, but non-veridical, naturalistic audio feedback of tapping strength (quiet, medium, or loud) affects finger tapping behavior for both real and virtual surfaces (Furfaro et al., 2015). Maximum acceleration values during tapping were significantly affected by sound: with quiet tapping sounds affecting acceleration values more than loud tapping sounds. When tapping the real surface, the quiet tapping sound resulted in the largest changes in acceleration over time (as compared with medium and loud tapping sounds), as well as the largest variations between individuals (Furfaro et al., 2015). Such findings were interpreted as participants trying to deal with the incongruence between action and sound (i.e., induced by the quiet tapping sound) by using various motor strategies such as trying to stop the hand or trying to put more force into their taps to increase the sound of the tap. For both real and virtual surfaces, the presence of audio-feedback (loud, medium, or quiet), resulted in an increase in the speed of participants’ tapping and a decrease in acceleration, with effects persisting for ∼1 min after the audio feedback had been removed.

A second study found that manipulating the frequency of non-naturalistic auditory feedback can alter touch behavior (i.e., haptics), both in terms of velocity of movement and the pressure used during the movement (Tajadura-Jiménez et al., 2014). The non-veridical sonification of tactile contact of the index finger with a wooden surface (as discussed above), was also found to alter real-time interaction with the surface. Specifically, when a sonified version of rice grains falling into a bowl was paired with touch, less finger pressure was applied when the sound was low frequency than medium or high frequency. By contrast, when a sonified version of a metal gong was paired with touch, participants performed slower movements for the medium frequency version as compared with the low or the high frequency sounds.

The auditory cues provided during interaction with an object also influence an individual’s movement. Varying the naturalistic contact sound when reaching to grasp an object influenced both reaching behavior and the contact points chosen when the information provided (auditory and visual) was incongruent (Castiello et al., 2010). The sounds produced by the fingers when grasping objects covered in different materials (aluminum, paper, string, wool) were recorded and then paired with the participants’ reach to grasp the object. When the auditory cues were non-veridical (i.e., incongruent), the grasping movement (movement duration, deceleration time, and grasp closing time) was significantly slower; in contrast, the grasping movement was significantly shorter when the sound provided happened to be congruent with the surface grasped. The information provided by the contact sound also shaped participants’ object contact behavior. The task used a stimulus object with differing material on the upper and lower half, and participants could choose to grasp either the upper or lower half. When varying sounds were provided, participants used the sound-congruent grasp the majority of the time (84%); e.g., if the sound related to paper, then fingers contacted the object on the half covered by the paper. Interestingly, previous research has shown that anticipated grasping motor action can be shaped by auditory cues (Sedda et al., 2011). Providing participants with the naturalistic sound that is made when placing a big or a small object on the table, results in an alteration to grip aperture that reflects the information provided by the sound (i.e., when the sound of small object was provided, a smaller grip aperture was used than when the sound of a large object was provided) (Sedda et al., 2011). Last, there may be differential effects of auditory cues on action dependent on the nature of the motor behavior. When surface exploration of various virtual surfaces via a haptic device was paired with different sounds (sounds of a fingertip touching sandpaper or a fingertip touching copy paper), there was no effect of sound on surface exploration time (Etzi et al., 2018). However, surface exploration clearly differs from a task such as an object grasp, the latter of which involves a more clearly defined behavioral goal.

Additional work supports the idea that auditory cues can influence motor behavior during goal-directed action (Tajadura-Jiménez et al., 2016). Auditory feedback was used to induce a feeling of an elongated arm [via spatial incongruence of the sound of finger tapping and actual finger location during tapping, as discussed above in Tajadura-Jiménez et al. (2012)]. This perceptual modification of an elongated arm resulted in changes in motor behavior (assessed via arm kinematics) during a reaching task (Tajadura-Jiménez et al., 2016). It was shown that participants’ movement patterns matched those expected if one’s arm was actually longer. That is, movement velocities during reaching were decreased following an adaption procedure that induced the feeling of an elongated arm and this behavior change did not occur when the sound of tapping was also temporally incongruent with real tapping during the adaptation procedure (control). Such findings suggest that auditory recalibration of body length can occur and this update to the internal sensory model has movement implications.

Consistent with the findings of motor anticipation-auditory interactions (Sedda et al., 2011) is recent work evaluating motor action in musicians (Drost et al., 2005). Providing naturalistic, but non-veridical, auditory input has been shown to induce errors in movement when past learnt associations exist between actions (movements on the piano) and their sensory outcomes (piano notes) (Drost et al., 2005). The pianists in one study were required to play a two-tone sequence (intervals) on the piano. They pressed a piano key (always E4) and were then given a visual note stimulus that instructed them as to which key they should press next. This visual note stimulus was paired either with congruent or incongruent auditory feedback. Responses in the incongruent condition were slower than in the congruent condition and the incongruent auditory interval often resulted in participants making an error. That is, the latter were more likely to play the perceived interval that the auditory cue provided, instead of the interval that the visual cue provided. That this effect did not occur in non-musicians underscores the role of learnt movement and auditory associations.

There is also evidence to suggest that changes to movement based on auditory cues may extend to gait (Bresin et al., 2010; Menzer et al., 2010; Tajadura-Jiménez et al., 2015a). Altering the frequency of naturalistic auditory cues, namely the sound produced by foot-steps, results in changes to gait (Tajadura-Jiménez et al., 2015a). Providing non-veridical low frequency sound foot-steps resulted in changes in foot pressure, with the heel remaining in contact with the ground for longer than during the high frequency condition (see Figure 3), which is largely consistent with an increase in the perceived body weight during the low frequency condition. In addition, there was a larger acceleration during the foot upward movement in the high frequency vs. low frequency condition, consistent with a perception of reduced body weight in the high-frequency condition. Interestingly, manipulating the temporal congruence of walking and naturalistic footstep sounds has also been shown to alter gait (Menzer et al., 2010). Specifically, walking speed was dependent upon the auditory delay period, with walking speed increasing *after* each stride (foot lift off to next step) and decreasing *at* each stride (during foot contact), with participants unaware of the changes to their gait. Similarly, providing non-naturalistic sounds via the interactive sonification of footsteps may influence people’s walking behavior (Bresin et al., 2010). The participants in one study were asked to walk with different emotional intentions (happy, sad, aggressive, or tender) while both non-veridical and veridical auditory inputs of the sound footsteps on different surfaces were provided (muddy ground/iced snow and wood panels/linoleum, respectively). Prototype active shoes that captured the foot pressure used to generate temporally congruent auditory cues were worn by the participants. There were no differences in foot-ground contact timing (heel-to-toe [for each foot] or heel-to-heel [between left and right foot] Inter-Onset-Interval; ms) based on the auditory cues. However there was a tendency for participants to walk more slowly when the footstep sound provided was that of ‘muddy ground’ and for participants to walk faster when the footstep sound provided was that of ‘iced snow’ (i.e., independent of the emotional walking intention, although these comparisons were not statistically significant.

The influence of auditory cues on chewing behavior was examined in two studies (Endo et al., 2016, 2017). As mentioned above, a “pseudo-chewing” sound was paired in real-time to a participant’s bite into various pureed and minced foods. These studies evaluated chewing behavior in terms of mastication intensity (calculated from the root mean square values for the EMG signal during mastication normalized against the maximum root mean square value over 12 conditions) and mastication rhythm (using the first peak frequency of Fast Fourier Transform (FFT) analysis with the FFT spectrum calculated from the rectified EMG signal). In both studies, mastication intensity and rhythm were not significantly influenced by the presence of the pseudo-chewing sound (Endo et al., 2016, 2017). Given that the pseudo-chewing sound was temporally matched to behavior (i.e., was accurate in terms of onset and offset of jaw closing) and that the EMG derived chewing sound was a real-time correlate of the actual chewing force used, this suggests that tactile aspects of food texture influence chewing behavior (vs. non-veridical auditory input that suggests ‘chewiness’ of food). However, it is also possible that accurate temporal and force feedback during chewing (as captured in the real-time pseudo-chewing sound) actually sustains identical chewing behavior. Thus, it would be interesting to explore the use of non-veridical auditory cues during mastication in terms of altered temporal congruency with movement or altered auditory intensity [dB] relevant to jaw closing force to see if influences on mastication behavior may become apparent.

Finally, the use of naturalistic, non-veridical auditory cues has also been shown to influence breathing (Murgia et al., 2016). Naturalistic and sonified (rising tones inspiration; falling tones expiration) breathing sounds were provided where the post-expiration pause was manipulated (altering breath frequency). Participants were more likely to spontaneously match their breathing pattern (evaluated using reduction of breath duration variability) to naturalistic breath sounds than to sonified versions of breathing sounds and to a no-sound control condition (Murgia et al., 2016).

### Interim Summary

When considered together, these findings support the view that auditory cues impact movement behavior, ranging from changes in overt limb movements to changes in breathing rate (a movement that is typically autonomically driven). That movement itself is altered by auditory cues supports the existence of a multisensory feedforward and feedback system during movement (Wolpert and Ghahramani, 2000). That is, when an efferent motor copy is generated, sensory information from numerous sensory sources, including audition, is used to determine whether or not the intended movement was completed and is used to update the internal model underlying movement (Wolpert and Ghahramani, 2000). Thus, evidence of movement changes when auditory input is added suggests that audition is a sufficiently reliable input (Ernst and Banks, 2002) to allow for the updating of movement representations. The multisensory feedback system used during movement is typically considered to include information specific to the body part – e.g., from touch, proprioception, and vision. The present findings support the view that auditory stimuli, particularly naturalistic sound, has a substantial contribution to dynamic modulations of movement. That recent work has shown that undetected auditory cues can have influences on voluntary movement (tapping speed and single taps following delay of the participant’s choosing), suggesting that decisions to act are amenable to unnoticed sensory influences from the surrounding environment (Schurger et al., 2017).

Such findings may also have important links to clinical conditions such as apraxia, where due to neural injury, people have impaired intentional action execution. Research by Pazzaglia et al. (2008) has shown that there are unique auditory linkages to apraxia. That is, people with apraxia are also impaired in recognizing sounds that are specifically linked to human actions. Intriguingly, this effect is specific to the type of apraxia and its motor impairment. Matching of buccofacial (mouth and face) action-related sounds to visual images was significantly better in people with limb apraxia than buccofacial apraxia, with the opposite found for those with limb apraxia. That impairments in processing human action-related sounds parallel impairments in the same actions provides understanding of mechanistic underpinnings, and or clinical challenges, of this condition.

## Conclusion and Directions for Future Research

Taken together, the literature reviewed here demonstrates that there is growing evidence that auditory cues can substantially modulate how we perceive our own body, its movement, and the environment as well as influencing both the emotional reaction to movement and the performance of the actual movement itself. That crossmodal effects on perception of the body and movement extend to the auditory domain highlight the relevance of exploring and characterizing these interactions further in future research. Such work has both important theoretical and clinical implications.

First, the fact that auditory cues can influence people’s perception of the things that they interact with, and the rated pleasantness of the experience, is critical to consider for VR and AR that involves human interaction. It has, for instance, been shown that having auditory feedback of interaction with clothing in an AR environment increases the immersiveness of the experience, with consumers spending an average of 30% more time engaging with the product and willing to pay more for it (Ho et al., 2013). Additionally, the sonification of objects and environments in AR/VR set-ups could be key to allow utilization of such technology by individuals with visual or tactile impairment. There have been promising results associated with the use of auditory cues to allow visually impaired individuals to experience 3D objects in AR (Ribeiro et al., 2012) and to create accurate spatial mental maps of an indoor environment using VR (i.e., not requiring an individual to be physically present in the environment) (Picinali et al., 2014).

Second, the implications of auditory crossmodal influences on movement are significant for fields such as exercise and athletics training, as well as for clinical rehabilitation. For example, there is evidence to suggest that providing auditory feedback in training can result in changes to gait when running (Tate and Milner, 2017), alterations in movement during simulated skateboarding (Cesari et al., 2014), improved hammer throw performance (Agostini et al., 2004), and changes in rowing speed (Schaffert et al., 2010). There is also evidence to suggest that sonification of movement enhances motor learning of a complex motor skill, such as rowing (Effenberg et al., 2016). In clinical populations, pairing auditory input with exercise has been shown to exert positive effects in motor rehabilitation for Parkinson’s disease and stroke populations (Pelton et al., 2010; Thaut and Abiru, 2010; Nombela et al., 2013), results in improvements in cardiovascular outcomes via device-guided breathing exercise (i.e., synchronization of breathing rhythm with acoustically delivered rhythms) (Gavish, 2010; Ekman et al., 2011; Mahtani et al., 2012), and increased movement and self-efficacy in those suffering from chronic pain (Singh et al., 2016). However, to date, the majority of the studies in these areas have used sound as a way to provide *veridical* feedback about the movement being performed. What has been relatively unexplored, at least until recently, is the use of non-veridical auditory cues to shape the performance and experience of movement, namely an individual’s perception of the movement that they have performed. Such work may well have important clinical and training implications. For example, many people with chronic pain are fearful of movement and avoid movement of the affected body part. Use of auditory cues that promote feelings of pleasantness during movement as well as feelings of reduced effort or increased ability would be well placed to augment movement in this population. Indeed, such preliminary work in the area of chronic pain is promising (Singh et al., 2016). Furthermore, a key challenge in training high-level athletes is to find new ways to maximize performance. Given this review’s findings of changes in movement evoked by non-veridical auditory cues, this suggests that manipulating audio feedback of self-generated movement during training could have important consequences that may result in improved athletic performance. Of relevance are those findings showing that altering visual markers of performance, such as through ghost riders in VR that provide non-veridical information about the athlete’s last training session, result in improved performance (Barathi et al., 2018). It is not unreasonable to speculate that similar training enhancement effects might also extend to those situations in which auditory input is supplied and altered. Critically however, the use of auditory cues in training or sporting environments needs to take careful consideration of sound volume given that it is well established that high exposure to loud noise can result in cumulative (and irreversible) hearing loss. Indeed, such concerns have arisen in recent media (Hallett, 2015) which highlights findings from the Australian National Acoustic Laboratories which show that most gyms are already too loud (and getting louder), exceeding safe noise levels (Beach and Nie, 2014).

Third, investigating the impact of auditory cues on perception of the body and movement is also well-placed to advance our understanding of, and potentially the treatment for, conditions in which impairments in body perception and ownership are present. For example, people with pathological limb pain report that their affected limb feels bigger than it actually is, with behavioral tests confirming this alteration (Moseley, 2005). Importantly, in this condition, visually altering perceived limb size, namely minimizing the limb, is an effective analgesic (Moseley et al., 2008). The findings of studies in this review, showing that auditory cues can dynamically modulate perceived body size (Tajadura-Jiménez et al., 2012, 2017b, 2018), raise the possibility that use of auditory cues, such as a descending/ascending pitch, may have similar effects on pain, particularly when combined with other sensory cues (multisensory). Indeed, multisensory visuotactile body resizing illusions have been shown to result in significantly more analgesia in people with painful knee osteoarthritis than visual illusions alone (Stanton et al., 2018). Perhaps auditory cues may also result in a similarly enhanced effect when paired with vision or touch. Recent pilot work has shown that altering the sound of footsteps (steps too loud, or too quiet by altering frequency) in people with complex regional pain syndrome (a rare pain condition, with co-occurring altered body perception) modulated body perception, pain, and gait patterns (Tajadura-Jiménez et al., 2017a). However, large variations in effect were present (i.e., high frequency sounds did not always result in people feeling lighter). The variation in auditory-induced perceptual change in this population seemed to depend on the type of body perception disturbance, raising the possibility of a relationship between body perception disturbance characteristics and the ability for sonic feedback to alter body perception (Tajadura-Jiménez et al., 2017a).

Moreover, an in-depth understanding of what contributes to body ownership is particularly relevant in conditions such as somatoparaphrenia, where individuals report that a body part no longer feels as though it is their own (Valler and Ronchi, 2009). It is interesting to consider whether using auditory cues, not just to cue spatial attention (which has been shown to be helpful) (Salvato et al., 2016), but rather, to encode body movement and touch might be useful in such conditions. Mirror visual feedback has been shown to result in temporary remission of somatoparaphrenia – hypothesized to occur because it allowed the participant to see her arm “from the outside” (a 3rd person perspective) (Jenkinson et al., 2013). Perhaps auditory information could be used to do much the same – hear your limb moving “from the outside.” Regardless of treatment implications, this review supports the view that auditory input is used in the formation of multisensory representation of the body, suggesting that auditory cues may be a helpful tool to explore impaired perception in conditions such as somatoparaphrenia.

Last, the theoretical implications of exploring crossmodal auditory contributions to movement-related activity are substantial. Understanding how auditory cues shape perception may provide important information concerning how we create internal models of our own body and of its movement through space. Future exploration into whether there are individual differences in the ability of auditory cues to modulate perception and movement would appear relevant given findings that prior experience (i.e., musical training) influences the ability of auditory cues to shift motor behavior (Drost et al., 2005). Indeed, understanding the limitations of auditory cues to shift perceptions, as seen in numerous studies of this review (Tajadura-Jiménez et al., 2016, 2018), would provide telling information with regard to how crossmodal input interacts within the framework of perceptual inference. For example, studies in this review have shown that auditory cues impacted perceived arm length but only when the sound of tapping was two times (but not four times) the normal sound based on reaching distance (Tajadura-Jiménez et al., 2012), and perception of body height was only influenced when the sound of a ball dropping was delayed by a factor of two (perceived to be taller) but not when the sound was advanced (Tajadura-Jiménez et al., 2018). Such nuances in effects of auditory cues on perception extend to affective valence (feelings of pleasantness). That incongruence between visual/proprioceptive/tactile and auditory input when providing feedback of movement evoked the feeling of unpleasantness (Furfaro et al., 2015) suggests a link between valence and the violation of expectations (i.e., motor prediction error). These results are consistent with sensory-motor incongruence elicited using visual illusions: incongruence can result in heighted pain (increased unpleasantness) in clinical populations (McCabe et al., 2007) as well as a feeling of pain and discomfort in healthy volunteers (McCabe et al., 2005).

One question of interest here is whether the neural mechanisms that underlie the perceptual modulation during movement differ based on the nature of the auditory cue. There is clear evidence that temporal and spatial properties of sensory inputs play a key role in whether information from unimodal signals are integrated into a single multisensory percept (Ernst and Bülthoff, 2004; Spence, 2011). However, what is less clear is whether contextual features, or meaning, of the auditory stimuli play a role in such integration. For example, how do auditory cues modulate movement perception when the auditory cue is one that co-occurs with movement, such as the rustling of clothes as we move? And do the underlying mechanisms of perceptual modulation differ from a situation where auditory cues that would not be expected during movement are present, for example, cues of ascending pitch that are associated with increased speed of movement or upwards movement? It is possible that the pairing of unexpected stimuli involves the engagement of top–down processes that modulate stimuli via different neural mechanisms, and/or result in a cognitive/decisional, rather than perceptual, modulation. Previous research suggests that top down features, such as attention, may contribute to differences in how auditory cues modulate perception. For example, when task-irrelevant auditory stimuli are paired either with attended or unattended visual stimuli, the processing of tones paired with attended visual stimuli began to differ from those paired with unattended visual stimuli (Busse et al., 2005). Specifically, differences in ERP waveforms, originating in the auditory cortex, were found approximately 200 ms after stimulus onset, suggesting that attentional processes used in the visual modality influenced processing of irrelevant stimuli in the auditory modality (Busse et al., 2005). In addition, prior experience plays a key role in perceptual modulation, through its integration with noisy incoming sensory input, and this suggests that top–down expectations may also impact the processing of auditory cues. For example, those with musical training are more affected by auditory cues when judging the perceived location of a musical tone that is provided in various horizontal locations around the body (Timmers and Li, 2016). Given that low pitches are typically perceived to originate from the left and high pitches from the right (Lidji et al., 2007), although see Pratt (1930) for opposite findings, it might be expected that auditory cues would impact sound location judgments more in those with prior musical experience. Indeed, in those with musical expertise, the strength of the relationship between actual location and judged location reduces, while the strength of the relationship between judged location and presented pitch increases (Timmers and Li, 2016). That such perceptual influences do not occur in those without musical experience suggests that prior knowledge and experience can influence the level of perceptual alteration. One might postulate that similar effects may be seen with movement-relevant activity such that different prior experiences or expectations may influence the ability of auditory cues (and the type of auditory cue) to impact perception.

The large body of findings that have been reviewed here support the need for further work to unravel the influences of auditory cues on body perception and movement-related activity. Clear future directions include exploring the link between auditory cues and both perceptions of movement and experienced pleasantness of movement given the wide reaching implications for VR applications as well as exercise training and clinical rehabilitation.

## Author Contributions

TS and CS contributed to the conception of the work, the analysis and interpretation of the data, drafting of the manuscript and critical revision of content, and review and approval of final manuscript for submission.

## Conflict of Interest

TS received funding from Eli Lilly Ltd. to cover travel and accommodation costs in September 2014; this was unrelated to the present topic area. The remaining author declares that the research was conducted in the absence of any commercial or financial relationships that could be construed as a potential conflict of interest.
